# Conformal Human–Machine Integration Using Highly Bending-Insensitive, Unpixelated, and Waterproof Epidermal Electronics Toward Metaverse

**DOI:** 10.1007/s40820-023-01176-5

**Published:** 2023-08-16

**Authors:** Chao Wei, Wansheng Lin, Liang Wang, Zhicheng Cao, Zijian Huang, Qingliang Liao, Ziquan Guo, Yuhan Su, Yuanjin Zheng, Xinqin Liao, Zhong Chen

**Affiliations:** 1 Department of Electronic Science, Xiamen University, Xiamen, 361005 People’s Republic of China; 2 Department of Engineering Mechanics, School of Naval Architecture, Ocean and Civil Engineering, Shanghai Jiao Tong University, Shanghai, 200240 People’s Republic of China; 3 Academy for Advanced Interdisciplinary Science and Technology, Beijing Advanced Innovation Center for Materials Genome Engineering, University of Science and Technology Beijing, Beijing, 100083 People’s Republic of China; 4 Beijing Key Laboratory for Advanced Energy Materials and Technologies, School of Materials Science and Engineering, University of Science and Technology Beijing, Beijing, 100083 People’s Republic of China; 5 School of Electrical and Electronic Engineering, Nanyang Technological University, Singapore, 639798 Singapore

**Keywords:** Carbon-based functional composite, Multifunctional epidermal interface, Property modulation, Addressable electrical contact structure, Conformal human–machine integration

## Abstract

Efficient and flexible interactions require precisely converting human intentions into computer-recognizable signals, which is critical to the breakthrough development of metaverse. Interactive electronics face common dilemmas, which realize high-precision and stable touch detection but are rigid, bulky, and thick or achieve high flexibility to wear but lose precision. Here, we construct highly bending-insensitive, unpixelated, and waterproof epidermal interfaces (BUW epidermal interfaces) and demonstrate their interactive applications of conformal human–machine integration. The BUW epidermal interface based on the addressable electrical contact structure exhibits high-precision and stable touch detection, high flexibility, rapid response time, excellent stability, and versatile “cut-and-paste” character. Regardless of whether being flat or bent, the BUW epidermal interface can be conformally attached to the human skin for real-time, comfortable, and unrestrained interactions. This research provides promising insight into the functional composite and structural design strategies for developing epidermal electronics, which offers a new technology route and may further broaden human–machine interactions toward metaverse.

**Supplementary Information:**

The online version contains supplementary material available at 10.1007/s40820-023-01176-5.

## Highlights

- The addressable electrical contact structure enables the multifunctional epidermal interface with an all-in-one function of sense, recognition, and transmission, which realizes high flexibility and high-precision touch detection.
- The multifunctional epidermal interface achieves superior waterproofness and is constructed enough thin to be bent freely, which is not as rigid, bulky, and thick as common interactive electronic device.
- The bending-insensitive characteristic facilitates accurate and stable human–machine interactions, which provides a key foundation for intelligent prostheses and super-soft robots.

**Supplementary Information:**

The online version contains supplementary material available at 10.1007/s40820-023-01176-5.

## Graphical abstract

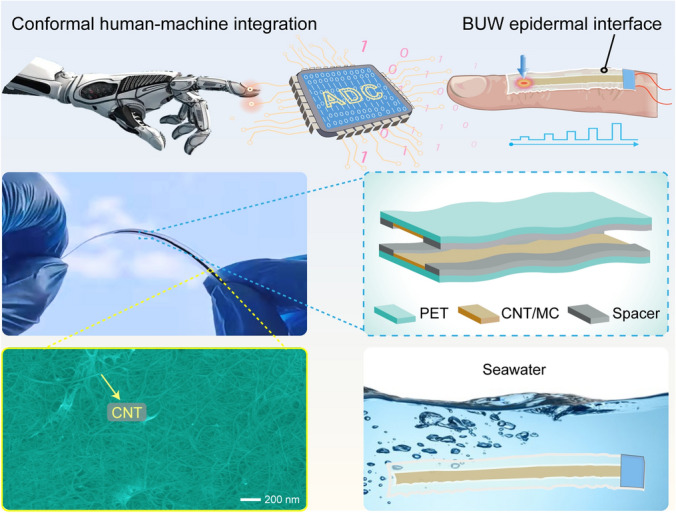

## Introduction

Interactive electronic devices (IE devices) in the metaverse enable users to enhance the quality of interactions and create real-time closed-loop feedback through an intuitive interface [1–7]. As if creating a virtual world, humans still can use their fingers and eyes to interact with the virtual world through IE devices, which makes the life of the avatar more real and the interaction more direct [8–11]. Metaverse reconstructs a new scene of the human–machine relationship, which gives an immersive interactive experience at any time as needed, such as exploring in a humid jungle or surfing on the wet sea. To resist humid surroundings, such as rain and sweat, IE devices are tightly encapsulated to obtain waterproofness [12–15]. This way generally makes IE devices to be rigid, bulky, and thick. Owing to the inherently soft human tissue surfaces, a large mismatch will be generated between IE devices and the human tissue surface [16–18]. To provide a smooth and relatively comfortable touch interaction, the IE devices need to be flexible and fit well on the skin surface [19, 20]. In reality, the current IE devices may still encounter malfunction due to bending deformation, making the sensing units out-of-operation or electrodes connection disconnected [21–23]. Thus, IE devices should have high flexibility so that they can be conformal with soft skin and continuously function stably even if they are deformed due to wearing [24–26]. Furthermore, the acquisition of high performance enables IE devices to accurately convert human intentions into computer-recognizable signals, which is critical to the breakthrough development of the metaverse.

Accurate and real-time information measurement using IE devices, such as stretchable transistors, haptic interfaces, and artificial skin [27–31], attached to the human body was crucial for human–machine interactions (HMIs). Through mutually non-overlapping capacitive signals, a flexible magnetic field sensor could identify the magnitude and direction of the magnetic field for contactless measurement and interaction [32]. To render entire IE devices flexible, efficient alternatives were to adopt soft functional materials to make sensing pixels flexible directly, which could be attached to the human skin as a touch operation platform [33–37]. To accurately recognize touch location, large-scale integrations of sensing pixels were common methods [38–41], which resulted in the overall stiffness of IE devices. To turn hard IE devices into flexible ones, alternatives were to design shape-variable conductive wires to connect sensing pixels by reducing pixel density [42–44]. Thus, high flexibility and high-precision touch detection seemed to be irreconcilable [45]. In addition, as sensing signals varied when flexible IE devices were mounted on the human body [23, 36], baseline offset or relationship redefinition between signals and instructions was generally needed while it was frustrating in HMIs.

Here, we introduce a highly bending-insensitive, unpixelated, and waterproof epidermal interface (BUW epidermal interface) to create conformal human–machine integration for free, wearable, and accurate interactions. The BUW epidermal interface mimics the structure and function of the mechanoreceptors in the biological touch sensory system, which can convert the touch information into the mechanosensitive signal for identification and differentiation. An addressable electrical contact structure is proposed to enable the BUW epidermal interface to precisely recognize touch location without large-scale integration of sensing pixels. The BUW epidermal interface is composited of ultrathin polyethylene terephthalate (PET) film, carbon nanotube (CNT), and methylcellulose (MC). By adopting a hierarchical assembly, the ultrathin PET film is served as an outside substrate, which endows the BUW epidermal interface with waterproofness and maintains the overall softness of the device. The excellent conductivity of the CNT makes the BUW epidermal interface possesses a rapid response time of < 8 ms and high-precision touch detection, which ensures accurate identification of users' control intent into stable and effective interactions. The MC provides a certain viscosity for the CNT to adhere well to the PET film, which guarantees that the signal transmission does not change even if the BUW epidermal interface is bent or deformed. As a proof-of-concept, this bending-insensitive characteristic is demonstrated by conformal integration of the BUW epidermal interface onto the people’s palms for virtually beating chime freely. Meanwhile, the unpixelated structural design strategy presented here facilitates the high-density touch sensing capability and the wide space of sensing area, of which the functional instructions can be programmable at will. Due to the practical scalability and versatility of the design principle, the BUW epidermal interface can be designed for special structures with different shapes for diverse interactive control toward the metaverse. The BUW epidermal interface not only proposes an unpixelated and waterproof structure for imitating the complicated biological touch sensory system but also innovatively exhibits the highly bending-insensitive characteristic to realize an artificial touch sensing system for broad relevance to conformally integrated electronics.

## Experimental

### Fabrication of the BUW Epidermal Interface

Considering that the thick polyethylene terephthalate (PET) could not be bendable. A thin PET film with the thickness of 20 μm (Foshan Wencheng Materials Co. Ltd) was selected as the substrate. Firstly, the PET film was washed with absolute ethanol and deionized water for 10 min to remove residue and dust. Secondly, carbon nanotube (CNT) dispersion with the volume of 8 mL (Beijing Dake Daojin Co. Ltd, purity of 0.2%, width of 1 ~ 2 nm, length of 5 ~ 30 μm) and methylcellulose (MC) with the weight of 0.01 g (Shanghai Titan Scientific Co. Ltd, viscosity of 15 mPa s) were mixed in the test tube and shaken well. The test tube was put into the ultrasonic cleaner (KQ5200DE, Kunshan Ultrasonic Instrument Co. Ltd.) for 30 min to make the CNT and MC (CNT/MC) mixture more evenly dispersed. Then, the edges of the PET film were firmly fixed horizontally on top of table using transparent tapes (No. 30028, Deli Company). Thereafter, four pieces of adhesive tapes (Scotch MagicTM Tape 810#, 3 M Inc.) were pasted on the fixed PET film to form a specified hollow pattern. Typically, the hollow area was 2 × 150 mm^2^. Around the hollow area, the size of the two longer pieces of adhesive tapes was 5 × 160 mm^2^. The size of the other two shorter pieces of adhesive tapes was 5 × 12 mm^2^. The CNT/MC mixture was dropped onto the hollow area and filled the hollow area fully. The processed PET film was naturally dried at room temperature for about 6 h. The drying could be accelerated using the oven at 50 °C for 50 min. After gently peeling off the four pieces of adhesive tapes, the CNT/MC-based sensitive material layer was formed and stably bonded with the PET film. The conductive wire was drawn out with copper foil tape (Dongguan Xinshi Packaging Materials Co. Ltd.) at one end of the long axis of the addressable sensing layer. The above experimental steps fabricated the basic upper part of the BUW epidermal interface, and another bottom part of the BUW epidermal interface was acquired by using the same preparation steps. The double-sided tapes (No. 5000NSWH, NITTO Inc.) were attached around the addressable sensing layers that served as spacers, of which the thickness was 160 μm, between the upper and bottom addressable sensing layers. The upper and bottom addressable sensing layers were face-to-face put together by the double-sided tapes and assembled into the BUW epidermal interface. Finally, the superfluous PET film beyond the spacers was removed. By adopting a hierarchical assembly, the PET film served as an outside substrate, which endowed the BUW epidermal interface with waterproofness. In addition, the double-sided tapes could make upper and bottom PET films tightly bonded together to prevent the CNT/MC-based sensitive material layer from moisture. It was considered that the biosecurity of CNT was in doubt. Therefore, the PET film was served as the outer material to protect the addressable sensing layer and effectively isolated CNT from contacting the human skin.

### Signal Process and Analysis

The signal processing system based on a microcontroller unit (MCU) of the Arduino Leonardo benefited from the increased versatility and upgradability of a microcontroller chip (ATmega32u4), which was often outperformed in terms of energy efficiency and real-time execution. The Arduino Leonardo based on ATmega32u4 had the capabilities to handle routine operations, which needed to achieve control of processing, transmission, and communication of data between the BUW epidermal interface and computer. It contained twenty-three digital Input/output pins for sending and receiving data, a 16 MHz crystal oscillator, a micro universal serial bus (USB) connection port, a power jack, an in-circuit serial programmable (ICSP) header, analog-to-digital converters (ADC) and a reset button. For application demonstrations, the BUW epidermal interface was connected in series with an auxiliary resistor (*R*_au_) (Fig. S1a). The junction between the BUW epidermal interface and the auxiliary resistor was connected to the analog input pin of the MCU. The auxiliary resistor was used in the driver circuit to divide voltage. This circuit converted the response resistance of the BUW epidermal interface (*R*_total_) into a voltage signal that could be recognized by the MCU. The corresponding formula of the response voltages (*V*_out_) was: *V*_out_ = *V*_CC_ / (*R*_total_ + *R*_au_) × *R*_total_, where *V*_CC_ was the applied voltage (5 V). The sampled analog output voltage of the BUW epidermal interface was converted to a digital ADC output ranging from 0 to 1023 (for the 10-bit ADC) using the internal analog-to-digital converter, which could be segmented into several sets by the MCU. To guarantee highly reliable virtual target object identification and control, the MCU directly communicated with the computer for the HMIs. For resistance characteristic tests by the digital multimeter, the circuit consisted of an external resistor (*R*_ex_) connected in parallel with the BUW epidermal interface (Fig. S1b). The external resistor was mainly used to prevent data acquisition delay, which was caused by excessive range conversion in the digital multimeter. In this situation, the test resistance (*R*_t_) was: *R*_t_ = (*R*_total_ × *R*_ex_) / (*R*_total_ + *R*_ex_). When there was no pressure on the BUW epidermal interface, the response resistance (*R*_total_) was none and replaced by 0. When the mechanical stimulation was applied to the BUW epidermal interface, the response resistance could be calculated by the formula: *R*_total_ = (*R*_ex_ × *R*_t_) / (*R*_ex_ − *R*_t_).

### Characterization and Measurement

All the surface micromorphology of the CNT was observed using the field emission scanning electron microscope (FESEM, SUPRA55 SAPPHIRE). Microscopic images of the touch area of the BUW epidermal interface were obtained by the digital optical microscope (ZWSP-4KCH, Medium Micro Innovation Technology Co. Ltd.). The customized actuator executed cyclic dynamic touching tests on the BUW epidermal interface (Beijing Times Brilliant Electric Technology Co. Ltd). The control panel of the programmable actuator was used to set the loading time and interval of the cyclic dynamic external mechanical stimulation. The standard force sensor (Bengbu Sensors System Engineering Co., Ltd., JHBS-1000G) was used to accurately calibrate the applied force. The response resistance of the BUW epidermal interface was recorded throughout the testing by digital multimeters (Proskit MT-1236 and Keysight 34465A). The thickness of the spacer was measured by the vernier caliper (K20K271592, GuangLu Digital measurement and control Technology Co. Ltd.). The electrical characterization of each BUW epidermal interface was measured using a source meter (Keithley 2450) with a two-probe mode.

## Results and Discussion

### Operation Principle and Design of the BUW Epidermal Interface

**Fig. 1:**
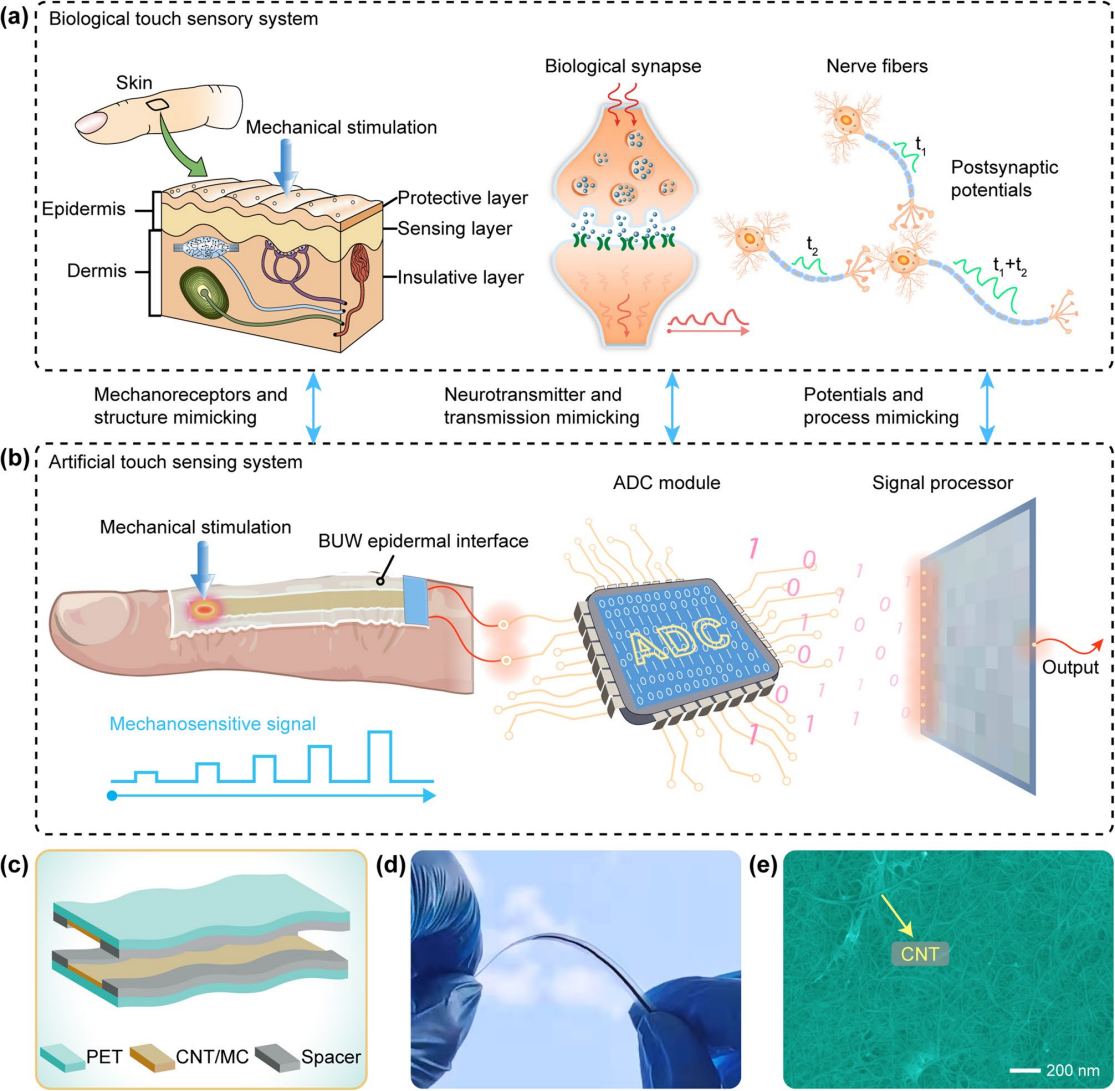
Biological touch sensory system and artificial touch sensing system. **a** Biological touch sensory system, including the skin, relevant mechanoreceptors, and neural system. **b** Artificial touch sensing system based on the BUW epidermal interface, ADC module, and signal processor. **c** Addressable electrical contact structure of the BUW epidermal interface composed of CNT/MC-based sensitive material layer, PET film-based protective layer, and spacer-based insulative layer. **d** Photograph of the BUW epidermal interface in the bending state, showing excellent bendability. **e** FESEM image of CNT/MC-based sensitive material layer, where the CNT was pointed out by a yellow arrow. (Color figure online)

The concept behind the biological touch sensory system and the artificial touch sensing system using the BUW epidermal interface is shown in Fig. 1. The biological touch sensory system detects external mechanical stimulation by converting them into receptor potentials using different types of cutaneous receptors (Fig. 1a), including Meissner corpuscle, Ruffini corpuscl, Merkel disk, and Pacinian corpuscle [46]. The receptor potentials are processed by synaptic transmission and spike generation. Different signals are gathered by nerve fibers. The resulting feedback conducts according to the brain, which judges the signals and makes decisions. Thus, the human body produces tactile perception for manipulating things by the corresponding feedback signals. To construct a system with an artificial touch sensing function, an artificial touch sensing system was designed that mimicked the human tactile recognition using the BUW epidermal interface, signal transmission, and processing system (Fig. 1b). The BUW epidermal interface sensed and recognized the external mechanical stimulation and transmitted the mechanosensitive signal to a microcontroller unit (MCU). Then, an analog-to-digital conversion (ADC) module in the MCU converted the analog signal into a digital signal. Finally, the digital signal was transmitted to a signal processor for making efficient decisions of HMIs. In the artificial touch sensing system, the BUW epidermal interface played a vital role in replacing various mechanoreceptors and generating mechanosensitive signals, which could sense and recognize the mechanical stimulation and transmit the mechanosensitive signal. The BUW epidermal interface was based on the addressable electrical contact structure. It consisted of two ultrathin PET films with the CNT/MC sensitive material, which were assembled in a mirror-symmetrical way. In the design, the BUW epidermal interface was thin enough to realize conformal integration with the human body. Figure 1c shows that the BUW epidermal interface was fabricated using the CNT/MC-based sensitive material layer, PET film-based protective layer, and spacer-based insulator layer. The microscopic images of the BUW epidermal interface clearly presented that the thickness of the addressable sensing layers was 27 μm (Fig. S2), where the thickness of the PET film-based protective layer was 20 μm (Fig. S3). Thus, the thickness of the CNT/MC-based sensitive material layer, which was part of the addressable sensing layer subtracted the PET film, was 7 μm. During encapsulation, the two addressable sensing layers were supported by a spacer-based insulative layer with the thickness of 160 μm. As the total thickness of the BUW epidermal interface was only 214 μm, which endowed the BUW epidermal interface with an excellent flexibility (Fig. 1d). The field emission scanning electron microscopy (FESEM) image presented the uniformly dispersed CNT/MC-based sensitive material layer, where the CNT was pointed out by a yellow arrow (Fig. 1e). It should be noted that CNT had good electrical conductivity but was not sticky. Since MC had good dispersion and viscosity, it could act as a binder and was mixed with CNT dispersion to make the CNT/MC-based sensitive material layer both conductive and sticky. Thus, the CNT/MC-based sensitive material layer could adhere well to the PET film no matter in the flat state or bending state. As a comparison, another epidermal interface with the thickness of 2 mm was fabricated. When being bent, the thick epidermal interface generated undesirable creases, which resulted in an inherent mismatch in functions (Fig. S4). This situation was similar to one of the common devices based on thicker and less elastic materials, which could not be used for conformal human–machine integration of free, wearable, and accurate interactions.

The designed addressable electrical contact structure and its equivalent circuit for the working principle of the BUW epidermal interface were explained in the electronic aspect (Fig. S5). The addressable sensing layer could virtually represent a series of resistive units, which was equivalent to the integration of multiple sensing pixels (Fig. S5a). The spacing area between the touch areas was similar to the air switch between the upper and bottom resistive units. When an external mechanical stimulation was applied at a touch location of the BUW epidermal interface, the corresponding air switch (e.g., S_2_ in Fig. S5a) closed. The BUW epidermal interface would generate a response resistance (*R*_total_), which was expressed as:

1 $$R_{{{\text{total}}}} = R_{{{\text{u1}}}} + R_{{{\text{u2}}}} + R_{{{\text{b1}}}} + R_{{{\text{b2}}}} + R_{{{\text{c2}}}}$$

where the *R*_u_ and *R*_b_ were the resistance of the virtual resistor units on the upper and bottom addressable sensing layers, respectively, and *R*_c_ was the contact resistance between the upper and bottom addressable sensing layers. Thus, the BUW epidermal interface could sense whether the external mechanical stimulation was applied. As the BUW epidermal interface was designed to be long-strip shape, any touch location of the BUW epidermal interface could respond to the external mechanical stimulation and generate a corresponding response resistance (Fig. S5b). When the external mechanical stimulations were applied at two touch locations of the BUW epidermal interface with the interval time Δ*t*, the corresponding air switches (e.g., S_2_ and S_4_ in Fig. S5b) would close and open sequentially. The corresponding but different response resistances of the BUW epidermal interface were generated to make a dynamic mechanosensitive signal. According to the response resistance of the mechanosensitive signal, the BUW epidermal interface could recognize the touch location, where the external mechanical stimulation was applied. The involving resistive units of the upper and bottom addressable sensing layers could transmit the electrical signal to the back-end circuit for processing and analysis. Therefore, the designed addressable electrical contact structure endowed the BUW epidermal interface with unpixelated sensing and recognition, which did not require large-scale integrations of sensing pixels. It greatly simplified the structure of the device so that the operation was efficient and convenient, thus reducing the difficulty of the fabrication process.

More prominently, as the BUW epidermal interface was ultrathin, the external mechanical stimulation could still be sensed and recognized even when the BUW epidermal interface was bent (Fig. S5c, d). Due to the presence of spacers, the upper and bottom addressable sensing layers were still separated even if the BUW epidermal interface was bent. Once the external mechanical stimulation was applied, the upper and bottom addressable sensing layers would contact, causing the BUW epidermal interface to generate a response resistance (*R′*_total_). The surface deformation (*ε*) caused by bending the BUW epidermal interface was calculated using this formula:

2 $$\varepsilon = h/2{r}$$

where *h* was twice the distance of the surface from the neutral plane and *r* was the curvature radius. Because the BUW epidermal interface was ultrathin, bending the BUW epidermal interface only made very small deformation of the upper and bottom addressable sensing layers (Fig. S5). This very small deformation would not further cause an observable change in the response resistance of the BUW epidermal interface. In this way, when the external mechanical stimulation was applied at the same touch location, the response resistance of the BUW epidermal interface would be almost the same, regardless of whether being flat or bent. Therefore, the response resistance of the BUW epidermal interface was insensitive to bending and could be used to accurately sense and recognize external mechanical stimulation. This bending-insensitive characteristic eliminated the need for baseline offset or relationship redefinition between signals and instructions, even if the BUW epidermal interface was deformed by wearing, which would facilitate comfortable and free HMIs.

It is worth noting that the biological mechanoreceptor possesses a threshold value for the input mechanical stimulation in a biological touch sensory system. The body cannot produce sensation when the input mechanical stimulation does not exceed the threshold value. In the artificial touch sensing system, the BUW epidermal interface also had a threshold value of external mechanical stimulation (Fig. S6). After the pressure of the external mechanical stimulation was larger than 4.5 kPa, the BUW epidermal interface could accurately sense and stably recognize the external mechanical stimulation. In the following tests, the pressure of the external mechanical stimulation was about or large than 6 kPa, unless otherwise noted. For common IE devices on standby, they were running at full power and the energy was always consumed regardless of whether external mechanical stimulation was applied, which would bring potential uncertainty or shortened life to long-term stable operation [12, 23]. However, when there was no external mechanical stimulation, the upper and bottom addressable sensing layers of the BUW epidermal interface were separated. Thus, the BUW epidermal interface did not consume energy on standby, which could improve the comprehensive energy utilization efficiency.

### Performance and Characteristic of the BUW Epidermal Interface

**Fig. 2:**
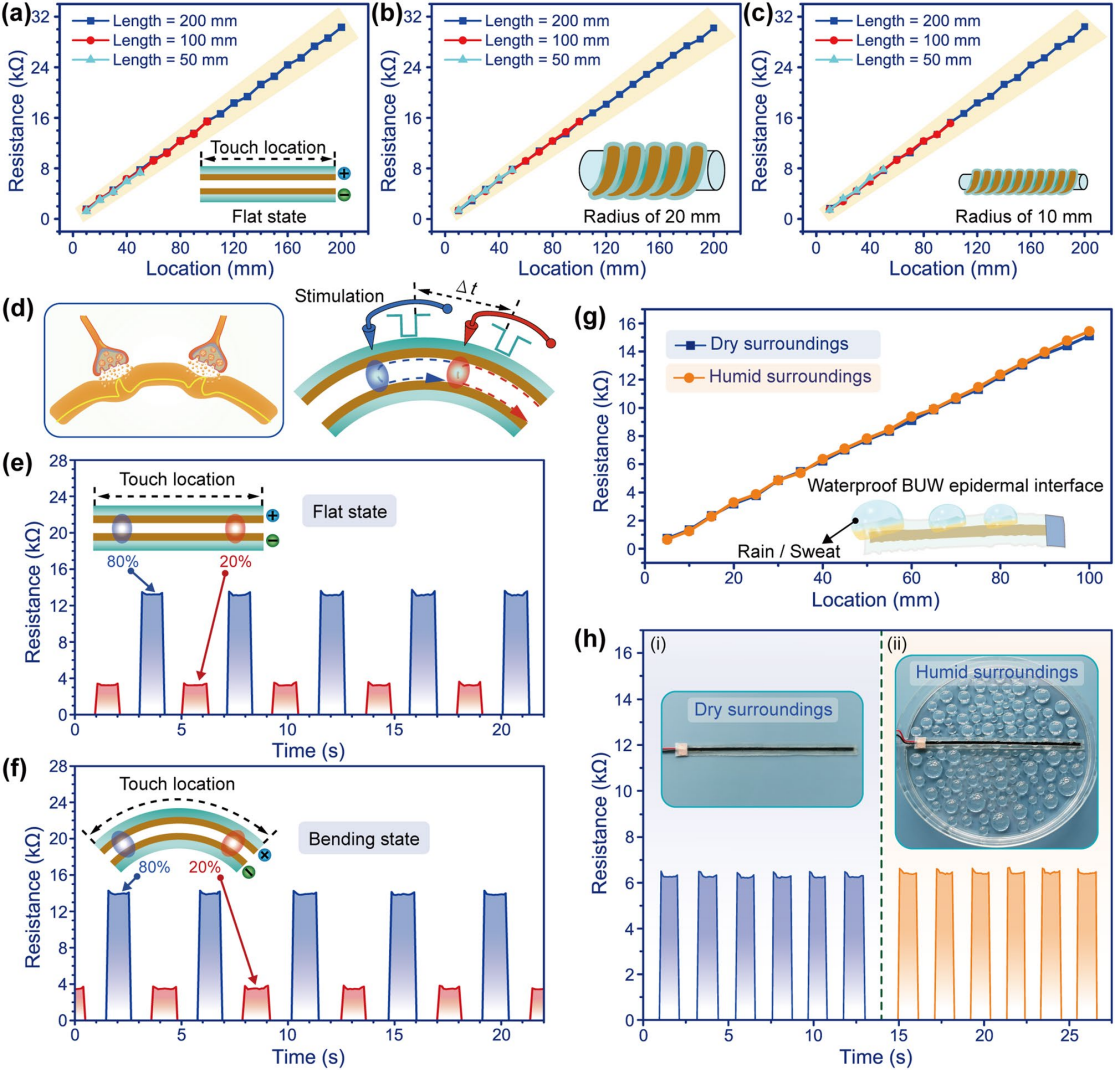
Bending-insensitive, spatiotemporal dynamic, and waterproof characteristics of the BUW epidermal interface. **a** Relationship between response resistance and touch location of the BUW epidermal interface with different lengths. **b**,** c** Change in response resistance versus touch location when the BUW epidermal interface was winded diagonally around cylinders with different radiuses. **d** Diagrams of two biological synapses and the BUW epidermal interface subjected to an external mechanical stimulation, which was applied at different location. **e**,** f** Repetitive responses of the BUW epidermal interface in the flat or bending state when applying the spatiotemporally dynamic stimulations. **g** Relationship between response resistance and touch location of the BUW epidermal interface in dry surroundings and humid surroundings. **h** Photographs and multiple cyclical tests of the BUW epidermal interface in (i) dry and (ii) humid surroundings

Figure 2a shows the change in the response resistance of the BUW epidermal interface when external mechanical stimulation was applied at different touch locations. It could be found that the response resistance of the BUW epidermal interface increased almost linearly with the change of the touch location. It was mainly because the response resistance of the BUW epidermal interface was affected by the involving resistance of the addressable sensing layers (Fig. S7), which was linear with the length of the addressable sensing layer. In addition, regardless of the different lengths of the BUW epidermal interface, such as 50, 100, or 200 mm, the linear relationships between the response resistance and the touch location were almost the same. The longer the effective working length of the BUW epidermal interface was, the more abundant the touch location became. To test the response resistance of the bending BUW epidermal interface, the BUW epidermal interfaces were winded diagonally around cylinders with different radiuses (Fig. 2b, c). It could be found that the response resistance was relatively linear with the touch location no matter how the radiuses of the cylinders are, which was important to accurately recognize the touch location. When touching the same location, the response resistance of the bending BUW epidermal interface was almost the same as that of the flat BUW epidermal interface. This was because the BUW epidermal interface was so thin that the bending strain was too small to change the response resistance. In a dynamical loading-unloading test, the mechanosensitive signals were also almost in the same change way no matter whether the BUW epidermal interface was in the flat or bending state (Fig. S8). This bending-insensitive characteristic ensured that the bending BUW epidermal interface could stably recognize the touch location as the case in the flat state. It eliminated the need for baseline offset or relationship redefinition between signals and instruction, which provided great advantages as a highly stable and flexible IE device to be mounted on the curved body surface for comfortable and unrestrained HMIs.

In the biological touch sensory system, even when the skin tissue was bent or deformed, the basic dynamic characteristic signals of external mechanical stimulation could still be transmitted through the biological synapses and the nerve fibers. Thus, a recognizable and differentiated dynamic logic in the neural network would be established to obtain spatiotemporal signals of different touch locations (Fig. 2d). In the artificial touch sensing system, the BUW epidermal interface could also possess the spatiotemporal dynamic recognition capability even if in the bending state. In the flat state, the response resistance of the BUW epidermal interface would rapidly rise up to a certain value and remained over time when an external mechanical stimulation was applied at a touch location, which was at 20% of the length (Fig. 2e). Until the external mechanical stimulation was released, the response resistance quickly disappeared. When the external mechanical stimulation was applied at another touch location, which was at 80% of the length of the BUW epidermal interface, the response resistance arose with corresponding magnitude for the recognition. Once the touch location was changed to the original one, the magnitude of the response resistance would be changed accordingly. In this way, the external mechanical stimulation at different times and touch locations could be accurately sensed and recognized by the BUW epidermal interface. The spatiotemporal dynamic stimulation could be also recognized by the bending BUW epidermal interface (Fig. 2f). By continuous and repeated mechanical stimulation, the response resistance could vary according to the different touch locations over time. When the external mechanical stimulation with the interval time of 1.02 s was applied at the relative locations at 20% and 80% of the length of the bending BUW epidermal interface, the corresponding response resistance in periods was generated and kept within a certain magnitude range. It could be found that the response resistance of the BUW epidermal interface was highly distinguishable from the spatiotemporal dynamic mechanical stimulation. The results manifested that the response signal could reflect well about the spatial and temporal feature of the external mechanical stimulation no matter whether the BUW epidermal interface was in the flat or bending state. The geometrically hierarchical response signal of the BUW epidermal interface realized the sensing and recognition function of an IE device consisting of numerous sensing elements, but its actual structure did not require so many sensing elements.

For the utilization of the BUW epidermal interface in the human–machine interaction system, the response time, response stability, and response frequency were also crucial factors to be considered. Since the BUW epidermal interface was thin enough, mechanical stimulation could cause the upper and lower layers to contact rapidly. Once the mechanical stimulation was removed, the upper and lower layers could be quickly separated, so the response time would be extremely fast. By applying external mechanical stimulation on the BUW epidermal interface, the rapid response time and recovery time of both < 8 ms could be tested, providing a significant advantage for the real-time feedback control system (Fig. S9). As the device’s structure was unified and the CNT/MC-based sensitive material layer was homogeneous, the sensing characteristics of each location of the BUW epidermal interface were the same. Therefore, the response time did not vary according to the touch locations. Due to the design of the addressable electrical contact structure, the BUW epidermal interface could realize unpixelated sensing and recognition (Fig. S10). It could be found that the change of the response curve was continuous. The unpixelated sensing characteristic meant that there was no independent pixel on the BUW epidermal interface so that any locations could be recognized well and correspondingly produce specific response. Comparing with the pixelated device, the BUW epidermal interface could be fully flexible and not affected by the wiring topology. In addition, only two electrodes were required to accurately sense and recognize the location, while most pixelated devices required many pixel routings with numerous electrodes. Furthermore, the cyclic mechanical stimulation (6 kPa and 1 Hz) was applied on the BUW epidermal interface for long-term operation analysis (Fig. S11). During a long cycle test (> 20,000 times), the response resistance was slightly changed (~ 9%). It was considered that the compression position and depth of the customized actuator slipped slightly as time. It could be found that the BUW epidermal interface was still capable of producing a response curve with regular undulations. Therefore, the BUW epidermal interface possessed excellent long-term stability and durability for potential wearable applications. Furthermore, different touch response frequencies were tested when the BUW epidermal interface was in the bending state (Fig. S12). The result showed that within a routine frequency range, although the frequency of the external mechanical stimulation changed, the response resistance of the BUW epidermal interface was relatively stable and there was no distortion or frequency loss. Therefore, the BUW epidermal interface could be used for stable and continuous touch interactions without re-correction in the signaling processes. All these tests manifested that the BUW epidermal interface possessed the potential for dynamic consecutive sensing and recognition of external mechanical stimulation, which could be attached on the various parts of the human body.

To consider potential applications in wet operating environments (e.g., humid jungle environment or surfing at sea), the waterproofness was a crucial characteristic. Since the upper and bottom addressable sensing layers were sealed together face to face and the electrodes were completely encapsulated with the double-sided tape, the designed BUW epidermal interface was endowed with excellent waterproofness and possessed superior reproducibility and good stability whenever being used in dry and humid surroundings. Figure 2g presented the relationship between the response resistance and the touch location in dry and humid surroundings. It could be found that the response resistance of the BUW epidermal interface remained almost constant in surroundings with different humidity conditions. Due to the strong adhesive force of the double-sided tape, the addressable electrical contact structure of the BUW epidermal interface effectively isolated the CNT/MC-based sensitive material layer from water or sweat. In this way, the BUW epidermal interface possessed waterproofness and flexibility, not as rigid, bulky, and thick as common waterproof IE devices. During a dynamic test, the external mechanical stimulation was applied on the BUW epidermal interface in dry and humid surroundings (Fig. 2h). Multiple cyclic tests were performed in the same touch location, which was well reflected by almost the same response resistance. In addition, even when being submerged in water, the BUW epidermal interface could still recognize the external mechanical stimulation and possessed good reproducibility (Fig. S13). This response resistances were almost unaffected whether the BUW epidermal interface was in dry or underwater surroundings. Thus, the back-end information processing did not need to be recalibrated. The results confirmed that the BUW epidermal interface had good waterproofness, which could continue to function even in underwater surroundings.

Common IE devices were designed with complex connection lines between pixelated sensing elements. They would be completely disabled when partially damaged, resulting in the collapse of the entire interactive system. Attributing to the addressable electrical contact structure, even if part of the BUW epidermal interface was cut, the two cuttable rest would still be able to sense, recognize, and transmit touch information (Fig. S14a). Since the BUW epidermal interface mainly consisted of CNT/MC-based sensitive material, PET film, and double-sided tape, all of which were cuttable, the as-fabricated device could be conveniently cut off with the scissor. After cutting, one section of the BUW epidermal interface consisting of the remaining conductive sensing layer and the electrodes still retained the functions. The other section consisting of the terminal portion and the re-added electrodes could restore the functions. The test result showed that after part of the BUW epidermal interface was cut off, the linear relationship between the response resistance and the touch location was almost identical to that of the original one (Fig. S14b). Therefore, the BUW epidermal interface could be easily cut into different lengths depending on the need for personalized HMIs. Due to the unpixellated working characteristic, the BUW epidermal interface could be virtually divided into multiple working segments for the needs of intelligent social HMIs. A dynamic cyclic mechanical stimulation was applied at the different virtual working segments of the BUW epidermal interface for the test (Fig. S15). The result indicated that each virtual working segment could well sense and recognize the mechanical stimulation.

### Multifunctional Interactive Applications of the BUW Epidermal Interface

**Fig. 3:**
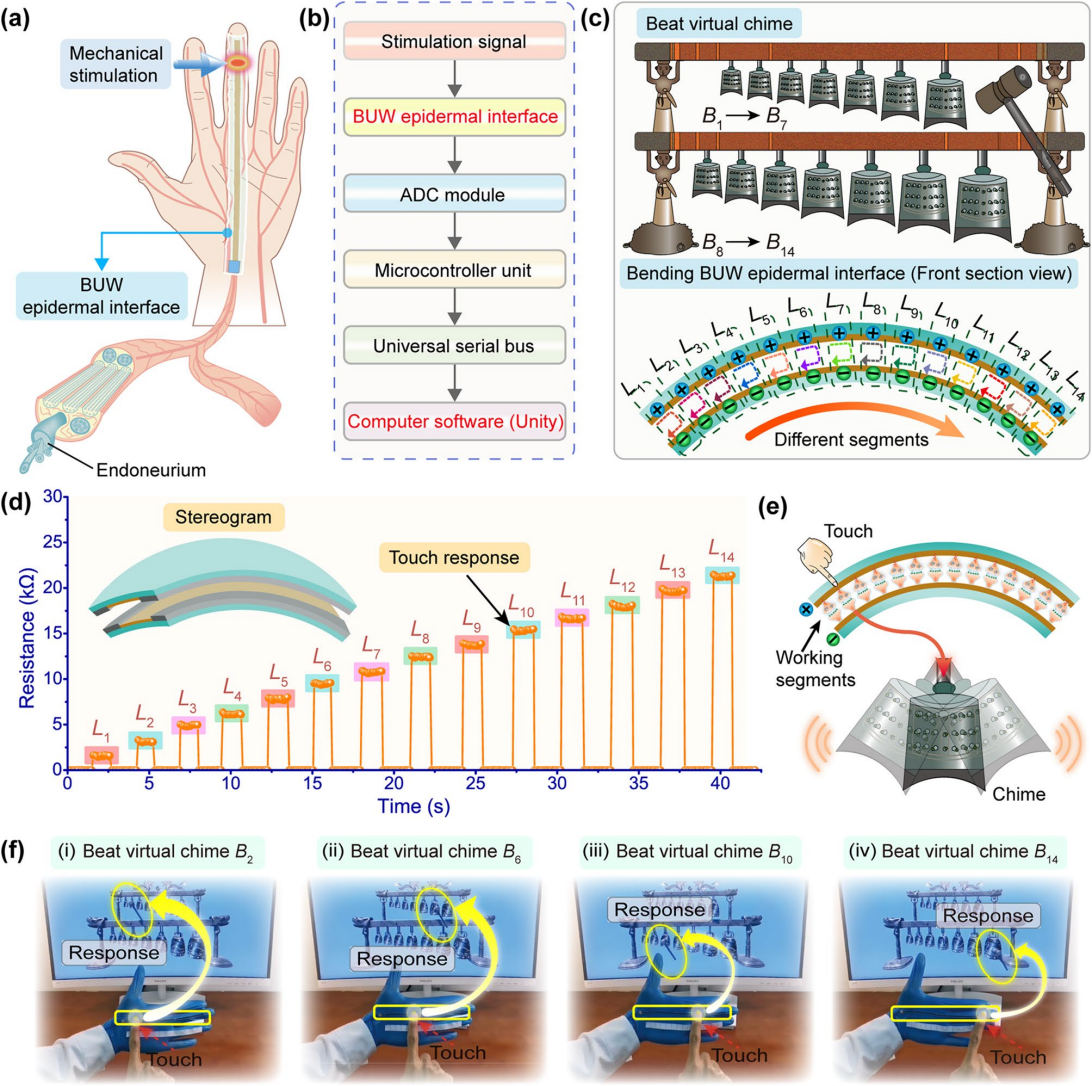
BUW epidermal interface for beating chime in the interactive VR system. **a** Schematic of the BUW epidermal interface installed on the palm. **b** Flowchart of HMI based on the BUW epidermal interface for the Unity application development. **c** Touching different working segments of the BUW epidermal interface corresponding to beating different chimes. **d** Typical response resistances when touching different working segments. **e** Touching a working segment of the BUW epidermal interface to trigger the corresponding chime. **f** Typical photos of beating the virtual chime by touching the BUW epidermal interface, where the yellow rectangles indicated the BUW epidermal interface fixed on the soft palm and the yellow ellipses indicated the beaten chime. (Color figure online)

With the rapid development of virtual reality (VR) technology, various types of IE devices were developed to enrich the entertainment experience of relaxation and decompression. The interactive signals would be fluctuating due to bending common IE devices to fit the curved surface of the soft human body so that inaccurate commands were generated to make a constrained experience. Herein, as a proof-of-concept, the BUW epidermal interface realized conformal human–machine integration to achieve the comfortable and unconstrained interaction of virtual chimes. Due to the highly flexibility, unique bending-insensitive characteristic, and excellent waterproofness, the BUW epidermal interface could be fixed on the curved surface of a soft palm to make the palm as an interactive platform for HMIs (Fig. 3a). An interactive VR system, which consisted of a BUW epidermal interface, an analog-to-digital conversion (ADC) module, a microcontroller unit (MCU), a universal serial bus (USB) interface, and computer software, was designed and developed (Fig. 3b). A linear BUW epidermal interface with an effective working length of 140 mm was designed and mounted on the palm to function. When the other finger touched the BUW epidermal interface, the touch location was deformed to make the specific upper and bottom addressable sensing layers contact to quickly generate a mechanosensitive signal. Next, the MCU received and processed the recognizable mechanosensitive signal. Finally, the signal was judged and transmitted to the Unity development application that controlled the virtual chimes through the universal serial bus. It was considered that the size of the pressing area would be different in different touches and the touching width of a finger was about 10 mm. In the demonstration, the linear BUW epidermal interface was virtually divided into fourteen working segments with a width of 10 mm, namely from *L*_1_ to *L*_14_ (Fig. 3c). Thus, touching a specific working segment of the BUW epidermal interface from left to right could accurately beat the corresponding chime, namely from *B*_1_ to *B*_14_. Figure 3d shows that the response resistances of the working segments were highly different from each other, which was beneficial to efficiently and consistently performing the HMI of beating chimes. When a working segment of the BUW epidermal interface was touched, a virtual hammer would move to the corresponding location to beat the chime in the VR interactive system (Fig. 3e). It was like that fourteen couples of virtual mechanoreceptors and synapses were embedded into the BUW epidermal interface, each of which could sense and convert external mechanical stimulation into the mechanosensitive signal. After abundant trials and tests, the addressable sensing layers were divided into specific intervals, allowing the BUW epidermal interface to accurately sense touch and recognize its location. For example, when a fingertip touched a working segment, the BUW epidermal interface could sense the touch and recognize the touch location so that the corresponding chime was triggered and belled. As a conceptual wearable verification, some typical examples of interactively beating chimes were conducted by touching the BUW epidermal interface, which was fixed on the soft palm (Fig. 3f). The BUW epidermal interface could bend and deform with the soft palm. As the bending-insensitive and wearable characteristic, the BUW epidermal interface could work normally without baseline compensation or signal verification. Even if the BUW epidermal interface was bent, it could still accurately sense and recognize each touch and stably trigger the belling of the corresponding chime. In addition, the BUW epidermal interface was capable of realizing no energy consumption when there was no external mechanical stimulation, which paved the way for long-term and effective use for entertainment activities in the VR interactive system. A vivid demo using the BUW epidermal interface to interact with the virtual chimes was presented in Movie S1.

**Fig. 4:**
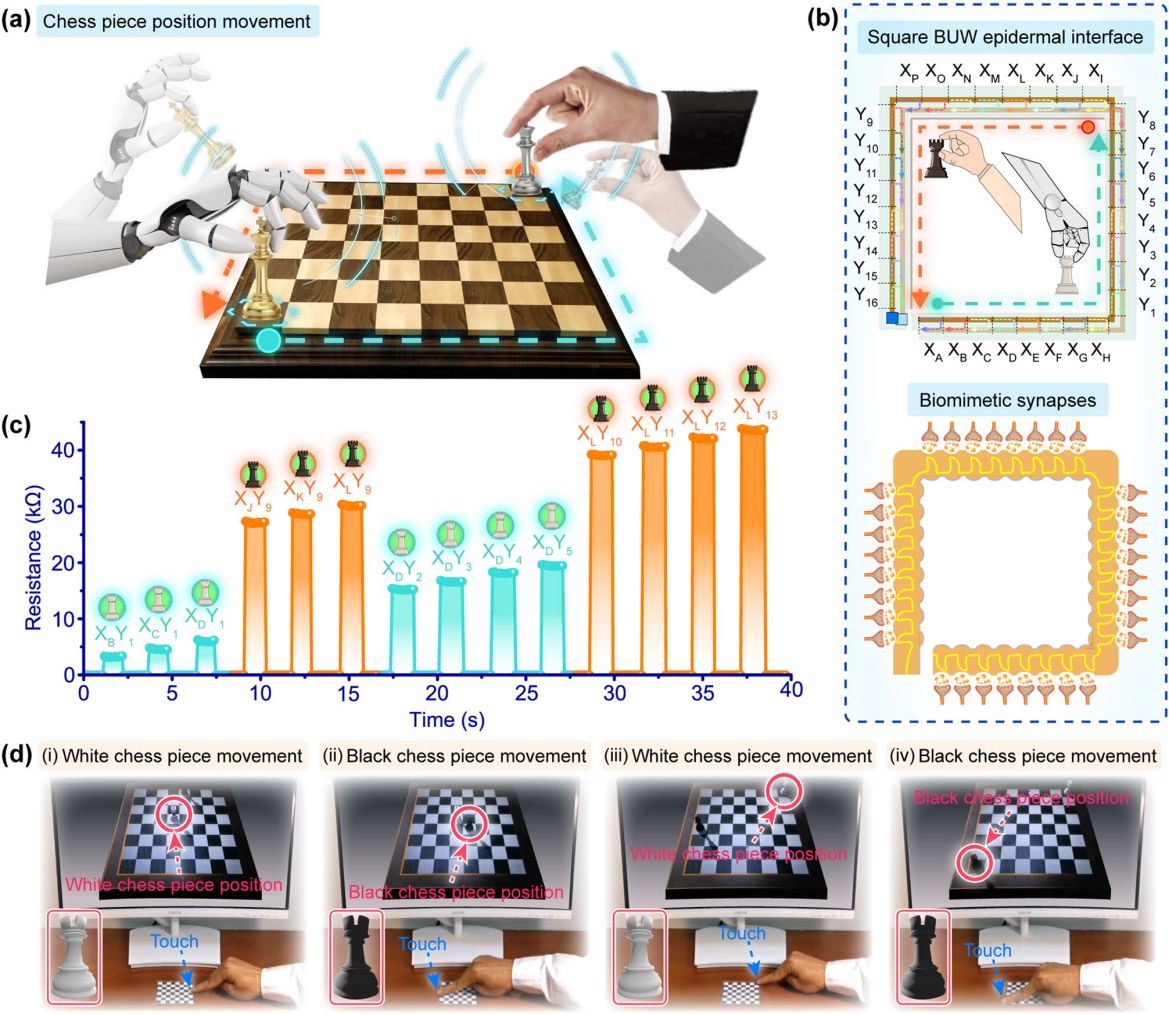
Square BUW epidermal interface for controlling chess piece position movement. **a** Diagram of the chess board and the movement trails of the white and black chess pieces. **b** Structure feature of the working segments of the square BUW epidermal interface and their corresponding biomimetic synapses. **c** Typical response resistances of the square BUW epidermal interface when touching different working segments. **d** Typical photos of different movement processes of virtual chess pieces by touching the square BUW epidermal interface, where red arrows indicated the chess piece positions and the blue arrows indicated the working segments of the square BUW epidermal interface. (Color figure online)

The designed addressable electrical contact structure greatly simplified the device structure and reduced the preparation process, thereby improving the scalability and versatility of the BUW epidermal interface. Thereupon, the BUW epidermal interface was designed into square, plus-shaped, and hexagon-shaped structures, which could be well applied to the corresponding interactive applications (Fig. S16). The representative demonstration of controlling chess piece positioning was introduced. Figure 4a shows the movement trajectories of the white and black chess pieces on a virtual chessboard in a competition between humans and machines. In the course of the chess piece movement, the white chess piece moved from the bottom left corner to the upper right corner and the black chess piece moved from the upper right corner to the bottom left corner. The white and black chess pieces in the VR space were created, which represented the controllable and programmable multivariant movement for chess piece positioning. Figure 4b presents the square BUW epidermal interface, which could be virtually divided into multiple working segments, and their corresponding biomimetic synapses. The highly regional differentiated recognition capability was realized by the square BUW epidermal interface (Fig. S17). The working segments at the bottom and right part of the square BUW epidermal interface were used to control the movement of white chess piece (the cyan arrow in Fig. 4b) and the other working segments were used to control the movement of black chess piece (the red arrow in Fig. 4b). The horizontal part controlled the horizontal movement of chess pieces and the other part controlled the movement of chess pieces along the vertical axis. When the finger touched the X_B_, X_C_, X_D_, Y_2_, Y_3_, Y_4_, and Y_5_ working segments of the square BUW epidermal interface, the response resistances of the corresponding working segments of the square BUW epidermal interface were rapidly generated (Fig. 4c). As a result, the white chess piece moved to the corresponding position on the virtual chessboard. Similarly, touching the X_J_, X_K_, X_L_, Y_10_, Y_11_, Y_12_, and Y_13_ working segments of the square BUW epidermal interface would make the black chess piece move to the corresponding position on the virtual chessboard. As a verification, this demonstration of the virtual white and black chess pieces’ movement by the square BUW epidermal interface was designed (Fig. 4d). In the developed interactive VR game-playing entertainment, touching different working segments would accurately make the virtual white and black chess pieces move to the corresponding position. A vivid demo using the square BUW epidermal interface to interact with the virtual chess pieces’ movement was presented in Movie S2. Owing to the facile square BUW epidermal interface, the human–machine interaction system based on touch not only provided a simplified and universal solution for capturing the human touch position but also introduced a strategy of avoiding the potential issues of the integration of numerous sensing units for the elimination of the complex interconnections and the signal crosstalk.

**Fig. 5:**
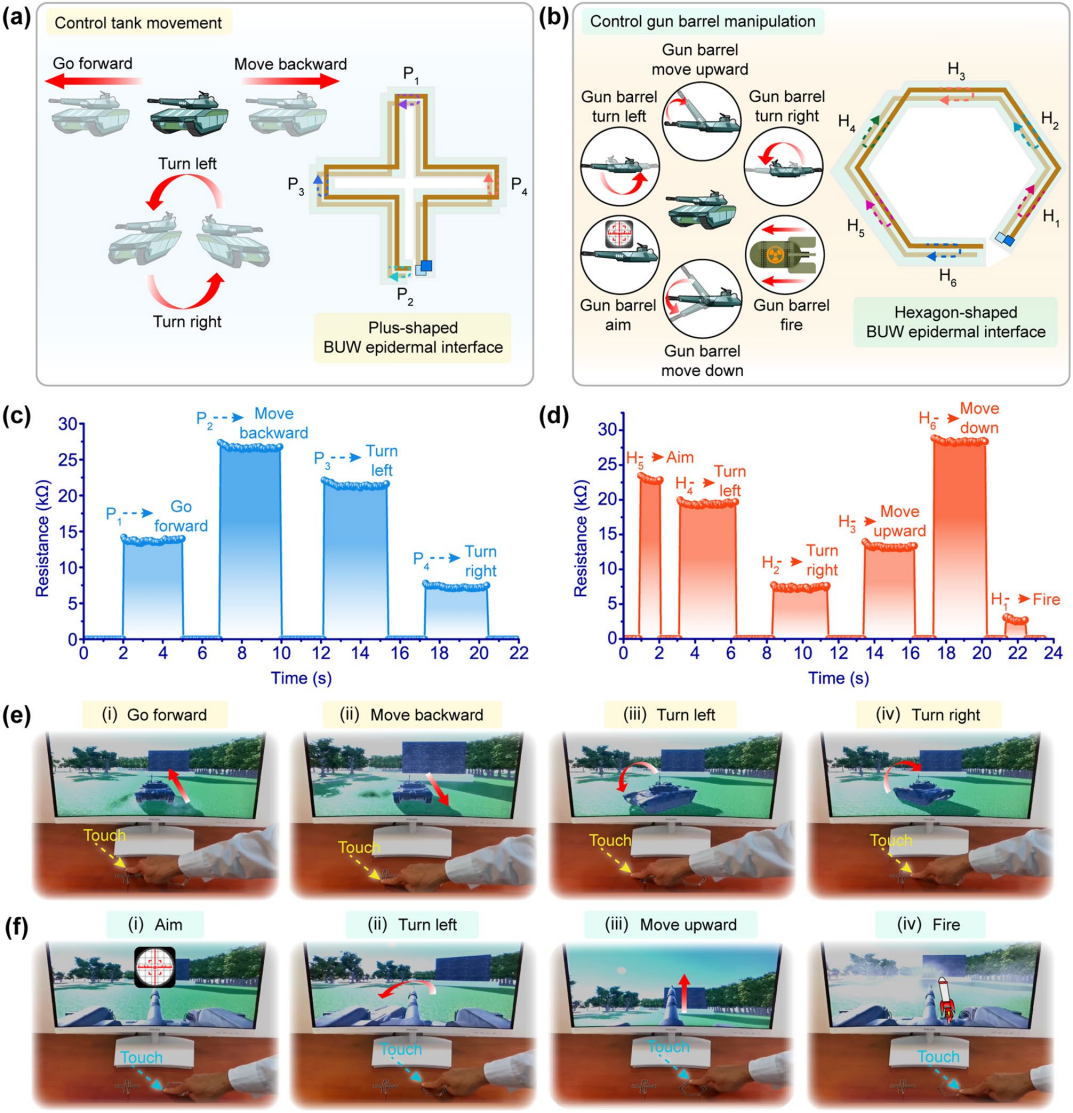
BUW epidermal interface with different shapes for controlling virtual tank movements and gun barrel actions. **a** Illustrations of four motion azimuth tracks in controlling tank movement and the plus-shaped BUW epidermal interface. **b** Illustrations of six operations in controlling the gun barrel and the hexagon-shaped BUW epidermal interface. **c** Response resistances of the plus-shaped BUW epidermal interface for virtual tank movements. **d** Response resistances of the hexagon-shaped BUW epidermal interface for gun barrel actions. **e**, **f** Typical photos of different processes of virtual tank movement using the plus-shaped BUW epidermal interface and gun barrel actions using the hexagon-shaped BUW epidermal interface

Various special shapes of the BUW epidermal interface were adapted to interactive entertaining applications to achieve an enhanced users’ experience. As an example, a plus-shaped BUW epidermal interface was designed and constructed as a plus-shaped touch panel to program a virtual tank movement (Fig. 5a). Four working segments of the plus-shaped BUW epidermal interface corresponded to four movement modes of the virtual tank, including going forward, moving backward, turning left, and turning right (the left image in Fig. 5a). Furthermore, to simulate the aiming manipulation of the gun barrel like a practical military exercise, a hexagon-shaped BUW epidermal interface was also designed and constructed to manipulate the gun barrel actions of the virtual tank (Fig. 5b). The manipulations of the gun barrel were controlled by the hexagon-shaped BUW epidermal interface, including aiming, turning left, turning right, moving upward, moving down, and firing (the left image in Fig. 5b). In the HMI of virtual tank movement, touching different working segments of the plus-shaped BUW epidermal interface caused the change in the response resistance so that controlling the corresponding virtual tank movement (Fig. 5c). By touching the specified working segments of the hexagon-shaped BUW epidermal interface, the response resistance would be generated quickly. In this way, the corresponding command would be issued to control the gun barrel actions (Fig. 5d). It could be found that these response resistances were significantly different regardless of the shape of the BUW epidermal interface, so the commands could be accurately sent according to the response resistances. More specifically, a demonstration of the typical virtual tank movement by the plus-shaped BUW epidermal interface was designed to be a training program for mastering the terrain during a specific military exercise (Fig. 5e). The primary demonstration of the typical virtual gun barrel by the hexagon-shaped BUW epidermal interface was conducted for practicing the aim and fire of the gun barrel (Fig. 5f). A vivid control of a virtual tank movement and the gun barrel actions by the BUW epidermal interface with different shapes was displayed in Movie S3, which was designed as a combat scheme of tank control to monitor terrain conditions and bombard the enemy. Due to the well consistency between the real touch sensing and the virtual object operations, the effectiveness of the actual training scenario program under complex human–machine interaction tasks could be greatly improved by the virtual practice.

## Conclusions

In the work, we have taken initial inspiration from the biological sensory system and made a breakthrough to construct a highly bending-insensitive, unpixelated and waterproof epidermal interface (BUW epidermal interface) for the artificial touch sensing system. CNT and MC were combined to achieve complementary advantages for preparing sensitive material with high electrical property and high stability. The addressable electrical contact structure with the satisfactory sensitive material was designed to enable the BUW epidermal interface with an all-in-one function of sense, recognition, and transmission. Without large-scale integration of sensing pixels, the BUW epidermal interface overcame the performance contradiction and realized high flexibility and high-precision touch detection. Due to the efficient addressable electrical contact structure, the BUW epidermal interface could achieve superior waterproofness and was prepared enough thin to be bent freely, which was not as rigid, bulky, and thick as common waterproof IE devices. Regardless of whether being flat or bent, the BUW epidermal interface could be conformably attached to the human skin as a touch operation platform with rapid response and recovery time of both < 8 ms and high durability of > 20,000 cycle tests. It eliminated the need for baseline offset or relationship redefinition between signals and instruction, which provided great advantages as a highly stable and flexible IE device to be mounted on the curved body surface for free, comfortable, and unrestrained HMIs. The BUW epidermal interface possessed the accurate spatiotemporal dynamic recognition capability like the one of the biological touch sensory system. The highly regional differentiated recognition capability and scalability, and versatility ensured that the BUW epidermal interface could be designed into as-needed shapes for diverse interactive control, including controlling chess piece positioning, the realistic tank movement, and the manipulation of virtual gun barrel. In particular, the BUW epidermal interface achieved the “cut-and-paste” character that allowed it to be corresponding size and mounted on the palm for diverse HMIs. The artificial touch sensing system with the BUW epidermal interface will enable things with an improved reproduction of tactile sense, which undoubtedly provides a major scientific and technological breakthrough for the accurate reproduction of human sense toward metaverse.

In the context of real-world interactive situations, the skin surface areas can not only be bent but also stretched. Therefore, for achieving the stretchability of the BUW epidermal interface, stretchable polymer substrates, such as poly(dimethylsiloxane), polyurethane, and Ecoflex silicone elastomer, may be viable alternatives. Furthermore, the combination of functional materials with stretchable substrate requires further exploration and research to realize stable signal output and recognition of the stretchable epidermal interface during the stretching process. The BUW epidermal interface based on the addressable electrical contact structure can effectively sense and recognize one touch point per time. In certain interactive applications, multi-touch sensing is also needed. It requires innovation in structural design and material exploration. Also, the simplicity and effectiveness of the device’s structure still should be deserved more attention. In addition, the exploration of body-integrated devices for real-time monitoring will dramatically benefit patients and remarkably improve their quality of life. Hence, one of the key challenges lies in the need for implantable and degradable materials. In the future, the combination of various functional materials and novel structures will enable the epidermal interface to be stretchable, multi-point touchable, implantable, and degradable.

### Supplementary Information

Below is the link to the electronic supplementary material.

Supplementary file1 (PDF 1882 KB)

Supplementary file2 (AVI 5005 KB)

Supplementary file3 (AVI 4149 KB)

Supplementary file4 (AVI 7101 KB)
