# Supplementary material for: Conformal Human–Machine Integration Using Highly Bending-Insensitive, Unpixelated, and Waterproof Epidermal Electronics Toward Metaverse
Source: Nanomicro Lett. 2023 Aug 16;15:199. doi: 10.1007/s40820-023-01176-5 (PMC10427580; doi:10.1007/s40820-023-01176-5)
Supplement: Supplementary file 1 — Supplementary file1 (PDF 1882 KB) [file 40820_2023_1176_MOESM1_ESM.pdf]

Supporting Information for

Conformal Human-Machine Integration Using Highly Bending-Insensitive, Unpixelated, and Waterproof Epidermal Electronics Towards Metaverse

Chao Wei[1], Wansheng Lin[1], Liang Wang[2], Zhicheng Cao[1], Zijian Huang[1], Qingliang Liao[3, 4], Ziquan Guo[1], Yuhan Su[1], Yuanjin Zheng[5], Xinqin Liao[1, *], Zhong Chen[1, *]

[1]Department of Electronic Science, Xiamen University, Xiamen 361005, P. R. China

[2]Department of Engineering Mechanics, School of Naval Architecture, Ocean and Civil Engineering, Shanghai Jiao Tong University, Shanghai 200240, P. R. China

[3]Academy for Advanced Interdisciplinary Science and Technology, Beijing Advanced Innovation Center for Materials Genome Engineering, University of Science and Technology Beijing, Beijing 100083, P. R. China

[4]Beijing Key Laboratory for Advanced Energy Materials and Technologies, School of Materials Science and Engineering, University of Science and Technology Beijing, Beijing 100083, P. R. China

[5]School of Electrical and Electronic Engineering, Nanyang Technological University, Singapore 639798, Singapore

*Corresponding authors. E-mail: liaoxinqin@xmu.edu.cn (Xinqin Liao), chenz@xmu.edu.cn (Zhong Chen)

Supplementary Figures

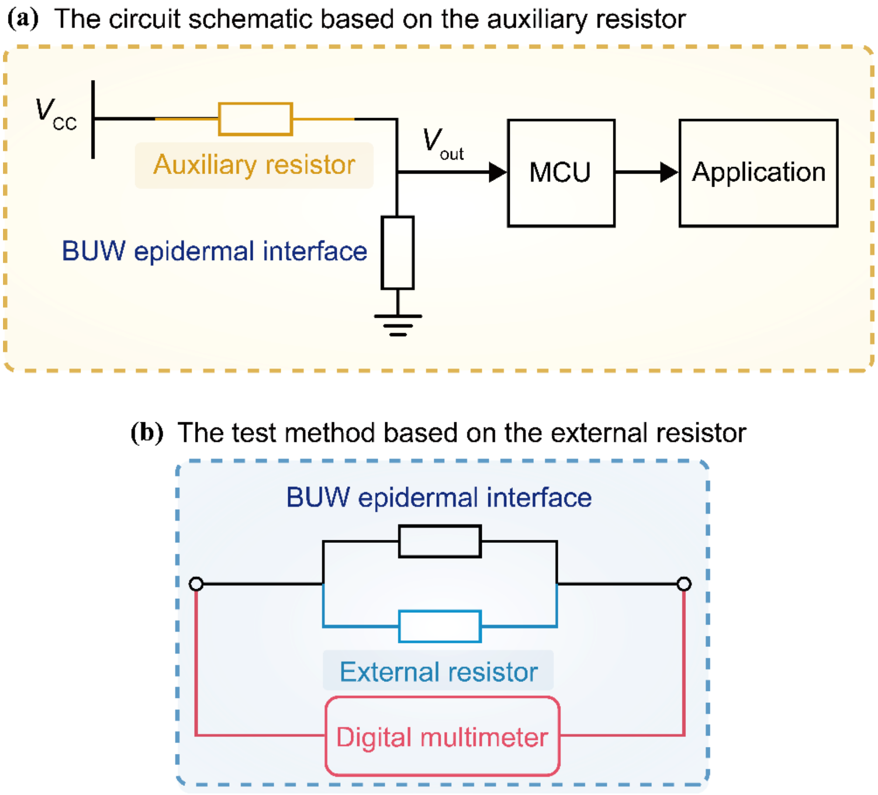

Fig. S1 Two different forms to connect resistors. **a** A drive circuit with an auxiliary resistor in series form for application demonstrations. **b** An external resistor in parallel form for resistance characteristic tests

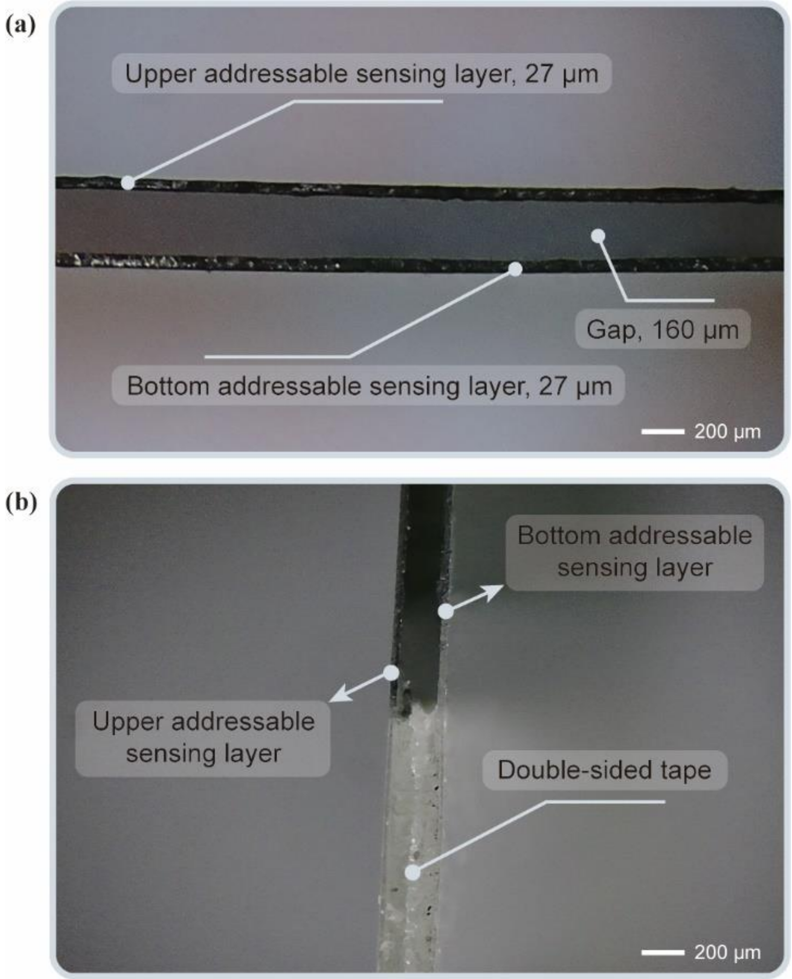

Fig. S2 Microscopic images of the BUW epidermal interface. **a** Microscopic image of the BUW epidermal interface in sectional front view, which clearly showed the upper addressable sensing layer, the bottom addressable sensing layer, and the gap between them. **b** Microscopic image of the connection between the touch area and the double-sided tape, which clearly showed the upper and bottom addressable sensing layers were separated from each other

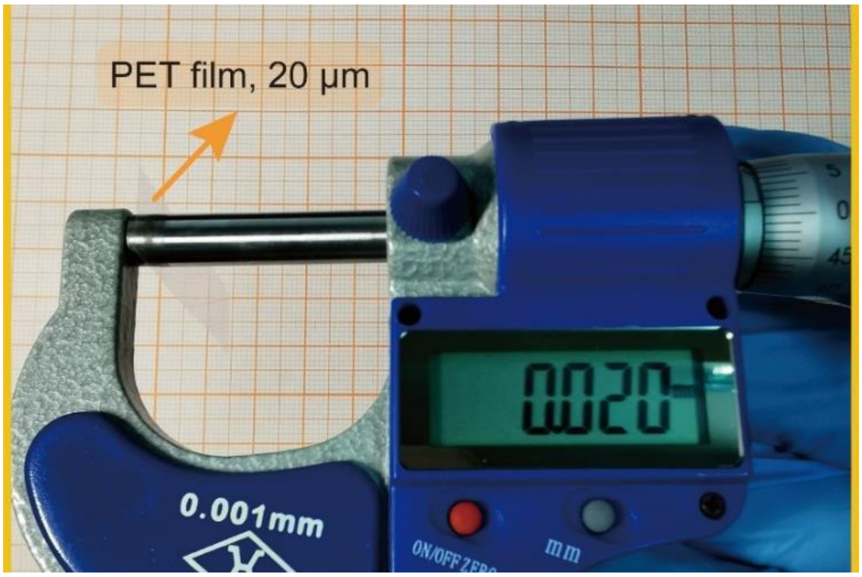

Fig. S3 The thickness of the PET film. The thickness of the PET film was measured using a micrometer caliper, showing a thickness of 20 µm

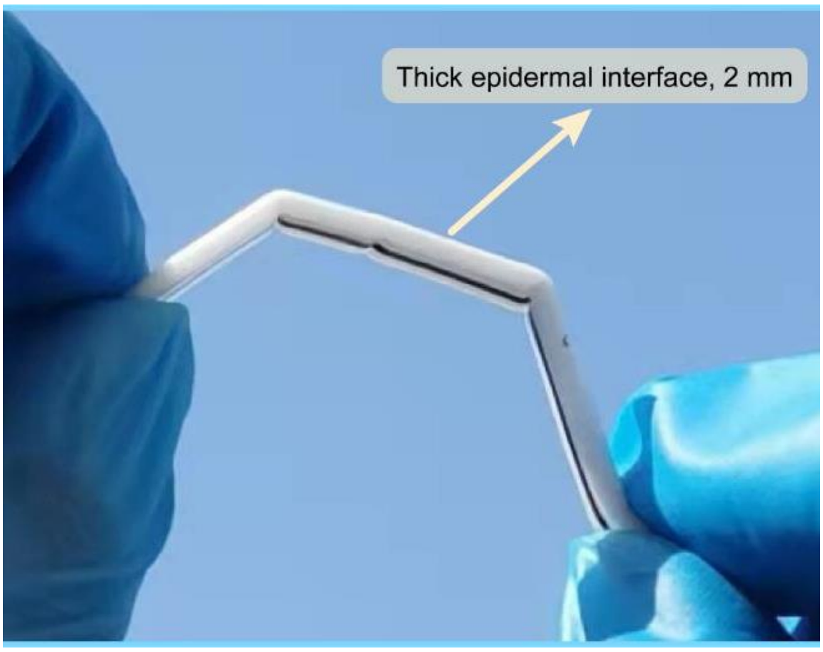

Fig. S4 Photograph of a thick epidermal interface with a thickness of 2 mm, showing the undesirable creases

The undesirable creases would result in an inherent mismatch in functions of the thick epidermal interface. This situation was similar to one of the traditional devices based on thicker and less elastic materials, which could not be used for conformal human-machine integration of free, wearable, and accurate interactions.

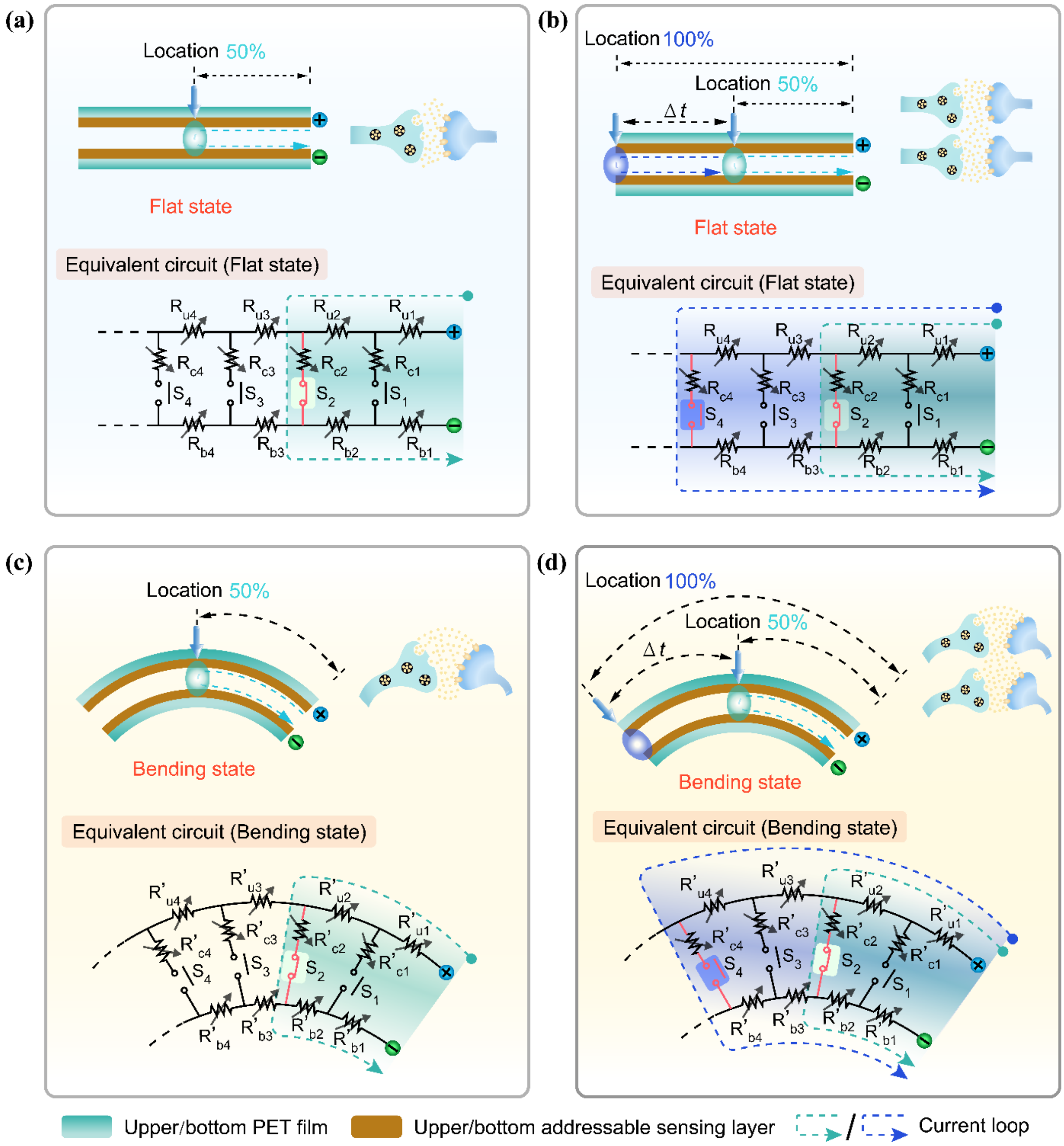

Fig. S5 Working principle of the BUW epidermal interface. **a** Top image: Touch applied on the 50% relative location of the BUW epidermal interface in the flat state. Bottom image: The corresponding equivalent circuit. **b** Top image: Touch applied on the 50% and 100% relative locations of the BUW epidermal interface in the flat state with the interval time Δt. Bottom image: The corresponding equivalent circuit. **c** Top image: Touch applied on the 50% relative location of the BUW epidermal interface in the bending state. Bottom image: The corresponding equivalent circuit. **d** Top image: Touch applied on the 50% and 100% relative location of the BUW epidermal interface in the bending state with the interval time Δt. Bottom image: The corresponding equivalent circuit

The surface deformation (ε) caused by bending the BUW epidermal interface was calculated using this formula: $\varepsilon = h / 2r$, where h was twice the distance of the surface from the neutral plane and r was the curvature radius. As an example, when the curvature radius was 20 mm, the deformation of the CNT/MC sensing material layer was $\varepsilon = (0.16 / 2 + 0.007) / 20 = 0.00435$. This deformation was very small. The resulting change in resistance hardly affected the resistance of the upper and bottom addressable sensing layers. Because the response resistance

of the BUW epidermal interface was mainly determined by the involving resistance of the upper and bottom addressable sensing layers, this very small deformation did not further cause an observable change in the response resistance of the BUW epidermal interface. In this way, when the external mechanical stimulation was applied at the same touch location, the response resistance of the BUW epidermal interface would be almost the same, regardless of whether being flat or bent. Therefore, the response resistance of the BUW epidermal interface was insensitive to bending and could be used to accurately sense and recognize external mechanical stimulation.

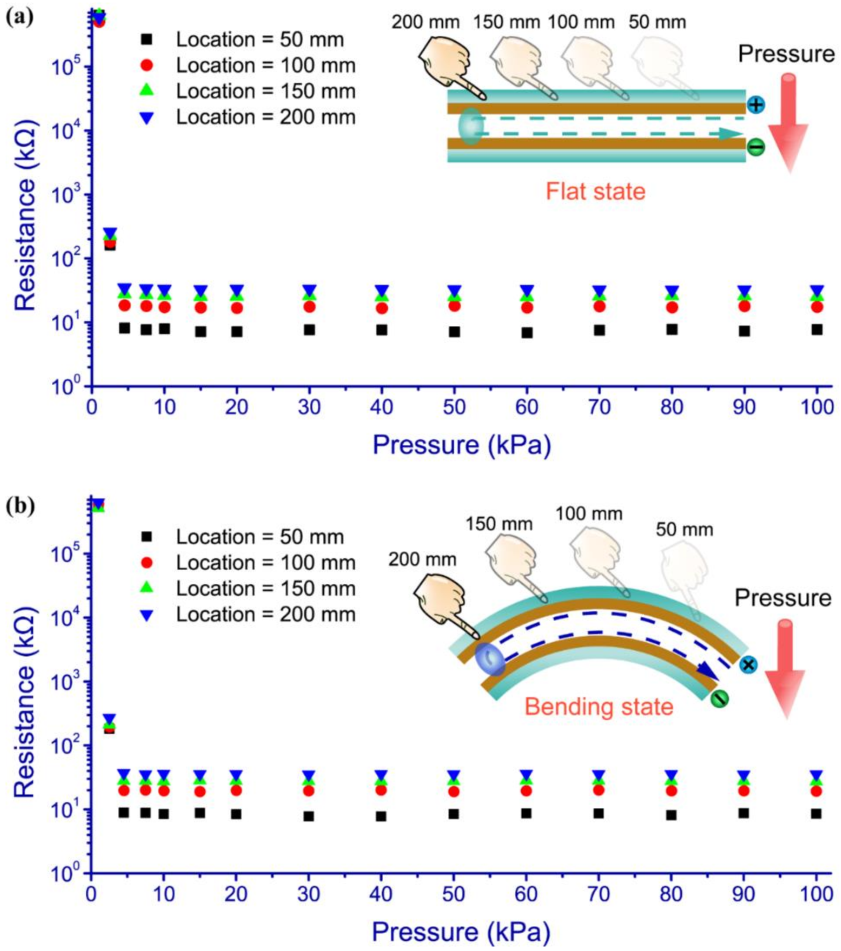

Fig. S6 Relationship of the pressure and response resistance for the BUW epidermal interface in the **a** flat and **b** bending state

The response resistance consisted of the resistance of the involving upper and bottom addressable sensing layers and the contact resistance between them at the touch location. The contact resistance was determined by the contact area, which depended on a certain pressure value of the external mechanical stimulation. The contact area caused by external mechanical stimulation varied from non-contact to close contact. When the applied pressure in the pressing area was less than the pressure threshold of the device, the size of the pressed area was less than the size of the saturation threshold on the contact area, so that the contact resistance was high. According to the electrical contact theory, the contact area became invariant until the pressure exceeded a certain value. In this work, when the pressure of the external mechanical stimulation was larger than about 4.5 kPa, the contact resistance was stable. Thus, when larger pressure was applied at a touch location, the response resistance of the BUW epidermal interface was only related to the touch location. The applied touch location could be recognized by the magnitude of the response resistance.

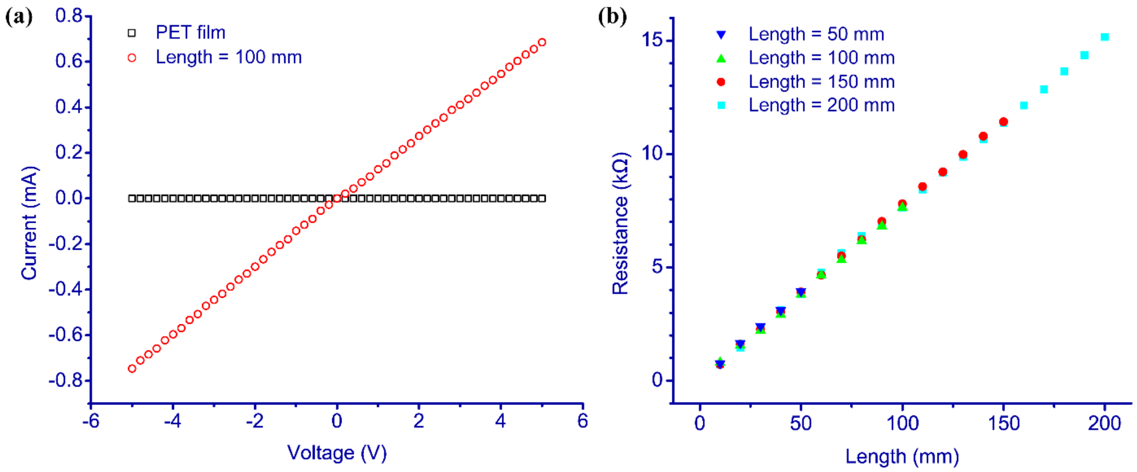

Fig. S7 Conductivity of different addressable sensing layers. **a** Current-voltage characteristic curve of the addressable sensing layer and PET film. **b** Resistance of different addressable sensing layers, of which the lengths were 50, 100, 150, and 200 mm, respectively.

The lengths of the PET film and the addressable sensing layer were 100 mm. It could be confirmed that the PET film was non-conductive. After the CNT/MC was coated on the surface of the PET film, the as-fabricated addressable sensing layer was endowed with electrical conductivity. The result from **b** indicated that the resistance of the addressable sensing layer was almost linear with the length. It reflected that overall the CNT/MC were uniformly coated on the PET film.

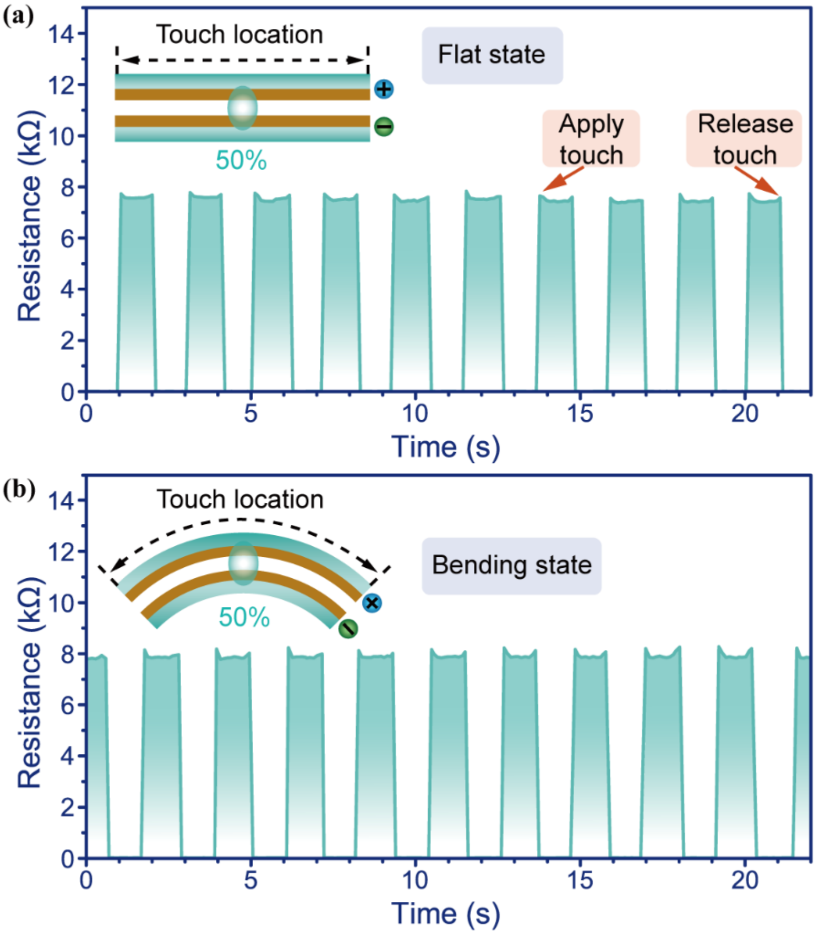

Fig. S8 Repetitive responses of the BUW epidermal interface in the **a** flat and **b** bending state responding to one type of mechanical stimulation

In the flat and bending state, the response resistance of the BUW epidermal interface would rapidly rise up to a certain value and remain over time when applying an external mechanical stimulation on one touch location (50% length). Until the external mechanical stimulation was released, the response resistance quickly disappeared. The rapid changes in electrical signal were similar to the excitation and inhibition of biological synapses to sense external mechanical stimulation. Continuous and repetitive mechanical stimulations with the interval time of 1.06 s would be reflected by the repetitive responses of the BUW epidermal interface, where the amplitudes of the mechanosensitive signals were almost unchanged even at the bending state.

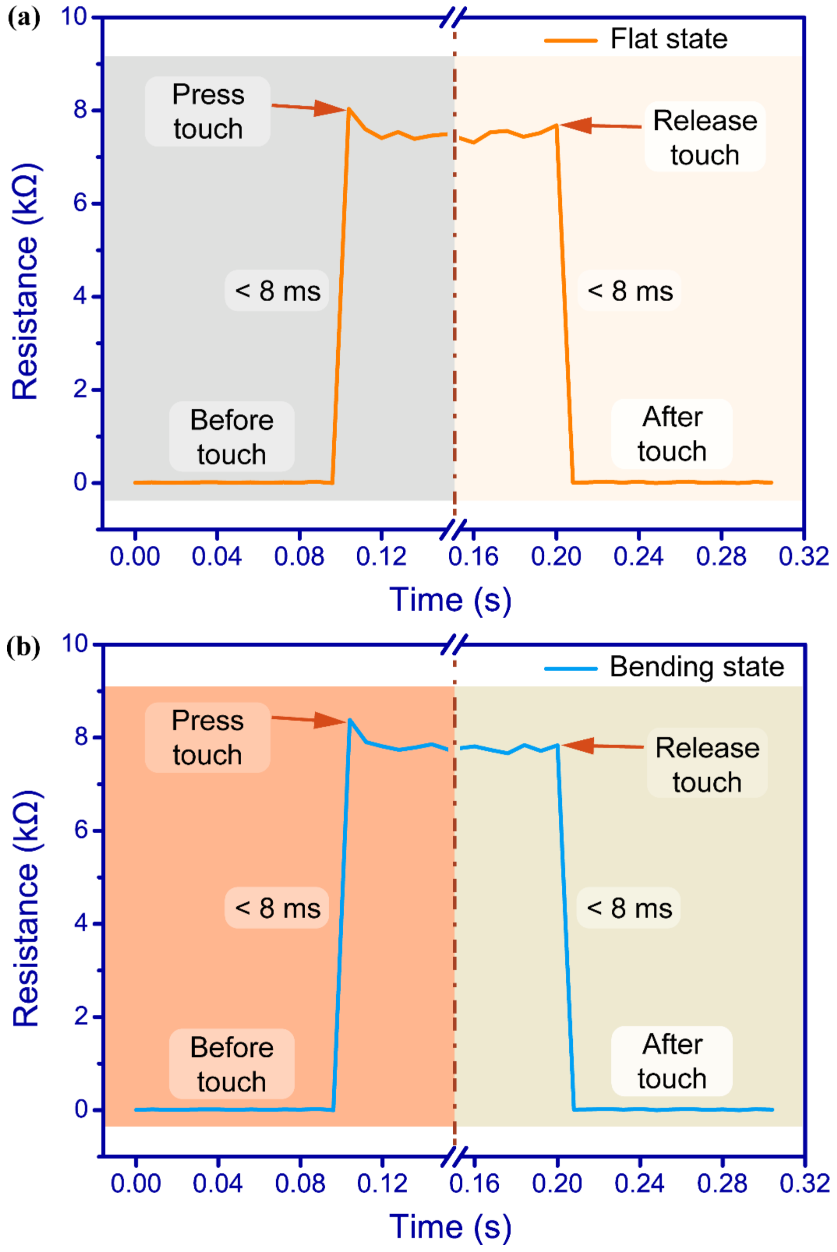

Fig. S9 Response time of the BUW epidermal interface in the **a** flat and **b** bending state

It could be found that the BUW epidermal interface responded rapidly (<8 ms) to the pressing touch and the touch signal disappeared rapidly (<8 ms) for the releasing touch. It provided a significant advantage for a real-time feedback control system.

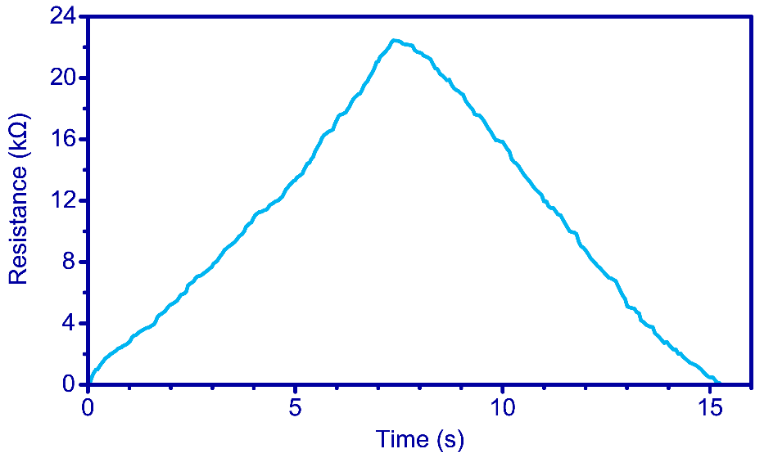

Fig. S10 Continuous slide test of the BUW epidermal interface

It could be found that the change of the result curve was continuous. Due to the design of the addressable electrical contact structure, the BUW epidermal interface could realize unpixelated sensing and recognition. During the tests, the edge of a card, of which the width was about 0.5 mm, was used to apply the mechanical stimulation. Therefore, the spatial accuracy of the BUW epidermal interface was less than 0.5 mm.

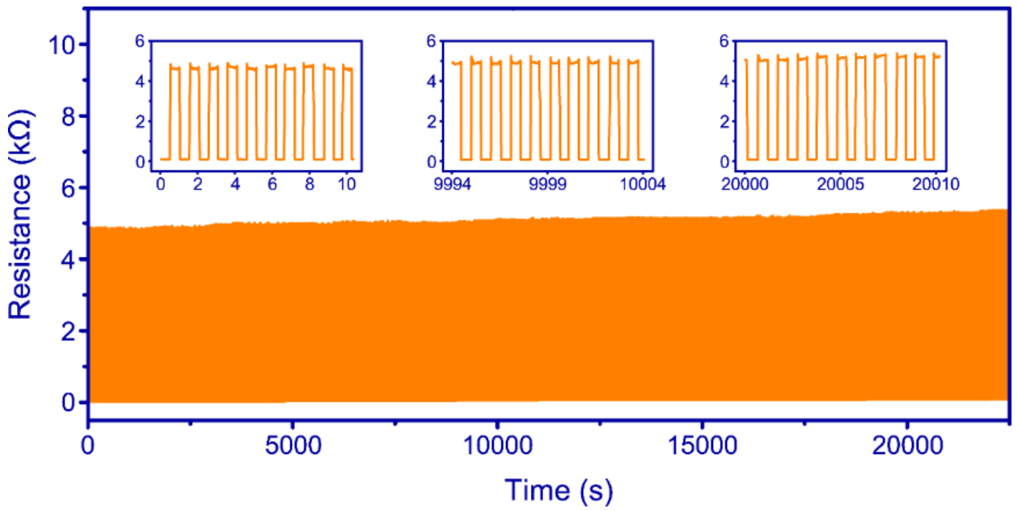

Fig. S11 Stability and durability of the BUW epidermal interface in the bending state

A cyclic test of over 20,000 times was conducted on the BUW epidermal interface by a customized actuator. The result showed that the response resistance was slightly changed (~ 9%) even for the 20,000 applying/releasing cyclic test when the BUW epidermal interface was in bending state. It reflected the prominent stability and durability of the BUW epidermal interface for long-term operation.

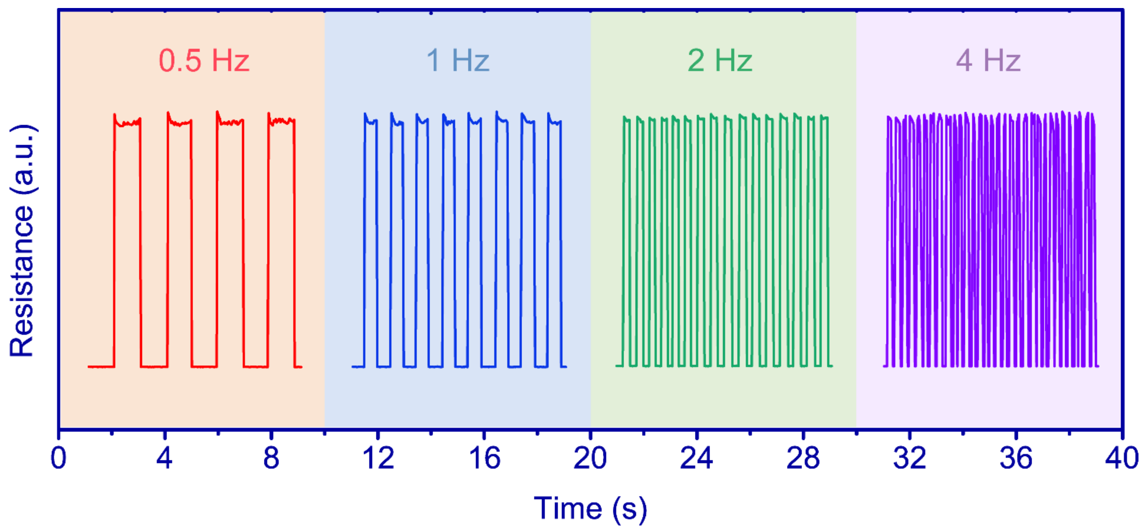

Fig. S12 Resistance response of the BUW epidermal interface under different pressing frequencies

The pressing frequency was increased from 0.5 to 4 Hz. It could be found that the resistance was almost unchanged, demonstrating the fast and stable response of the BUW epidermal interface.

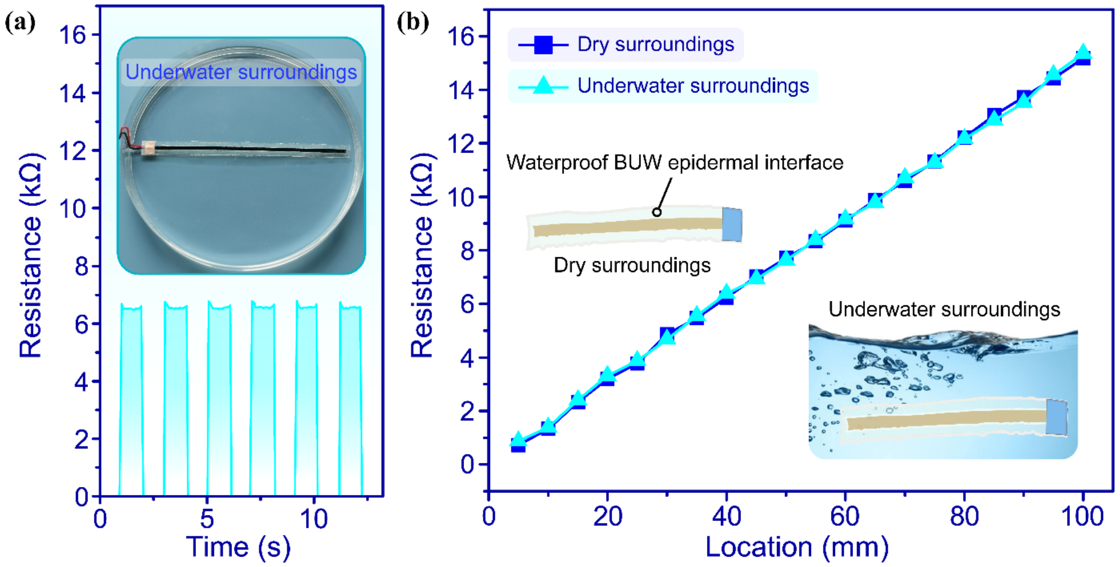

Fig. S13 Waterproofness tests of the BUW epidermal interface. **a** Photograph and dynamic cyclic test when the BUW epidermal interface being submerged in water. **b** Relationship between the response resistance and the touch location of the BUW epidermal interface in dry and underwater surroundings

It could be found that the response resistance of the BUW epidermal interface remained almost constant in dry and underwater surroundings.

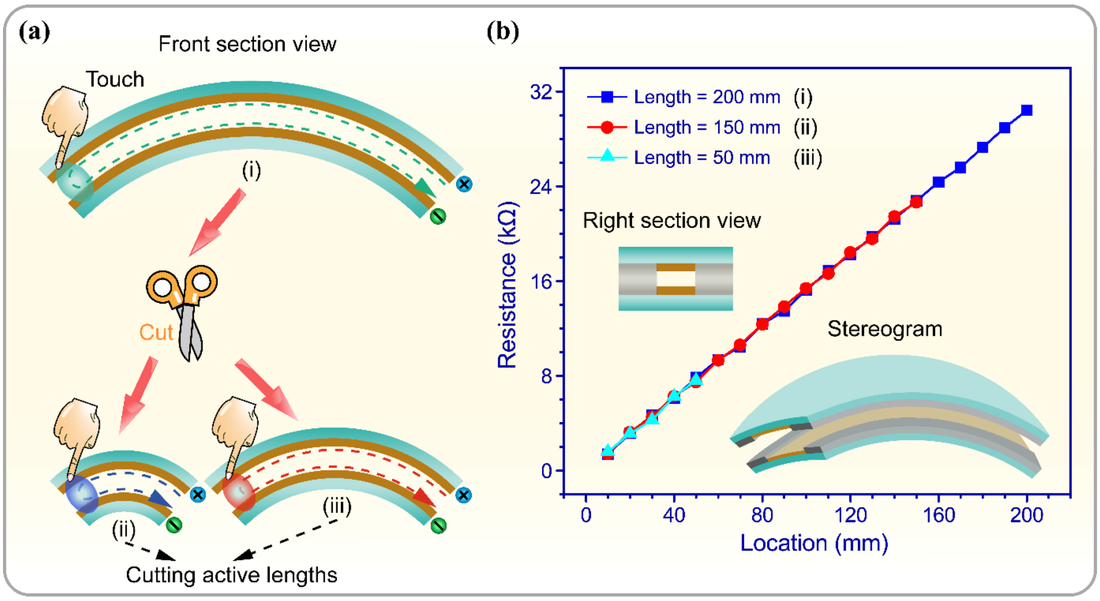

Fig. S14 Cuttability of the BUW epidermal interface. The BUW epidermal interface in the bending state could still work effectively after cutting. **a** Schematic of the cuttability. **b** Relationship between the response resistance and the touch location before and after the BUW epidermal interface was cut off

Since the BUW epidermal interface mainly consisted of CNT/MC, PET film, and double-sided tape, all of which were cuttable, the as-fabricated device could be conveniently cut off with the scissor. After being cut, one section of the BUW epidermal interface consisting of the remaining conductive sensing layer and the electrodes still retained the functions. The other section consisting of the terminal portion and the re-added electrodes could restore the functions. The test result showed that the linear relationships between the response resistance of the touch location were almost identical to that of the original one.

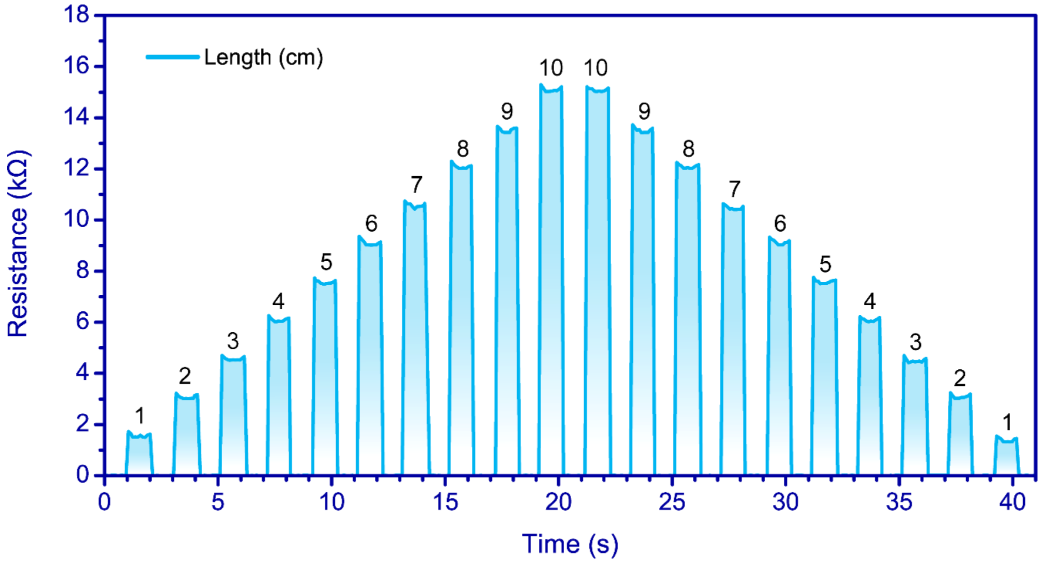

Fig. S15 Sensing characteristics of the BUW epidermal interface in the bending state

The dynamic cyclic tests were applied to the BUW epidermal interface in the bending state to demonstrate the sensing characteristics of the BUW epidermal interface. Response resistance for touching the sensing segments forth and back from 1 to 10 cm of the effective length of the BUW epidermal interface with the lasting time of 1 s. The result indicated that each virtual working segment could well sense the touch location of the mechanical stimulation.

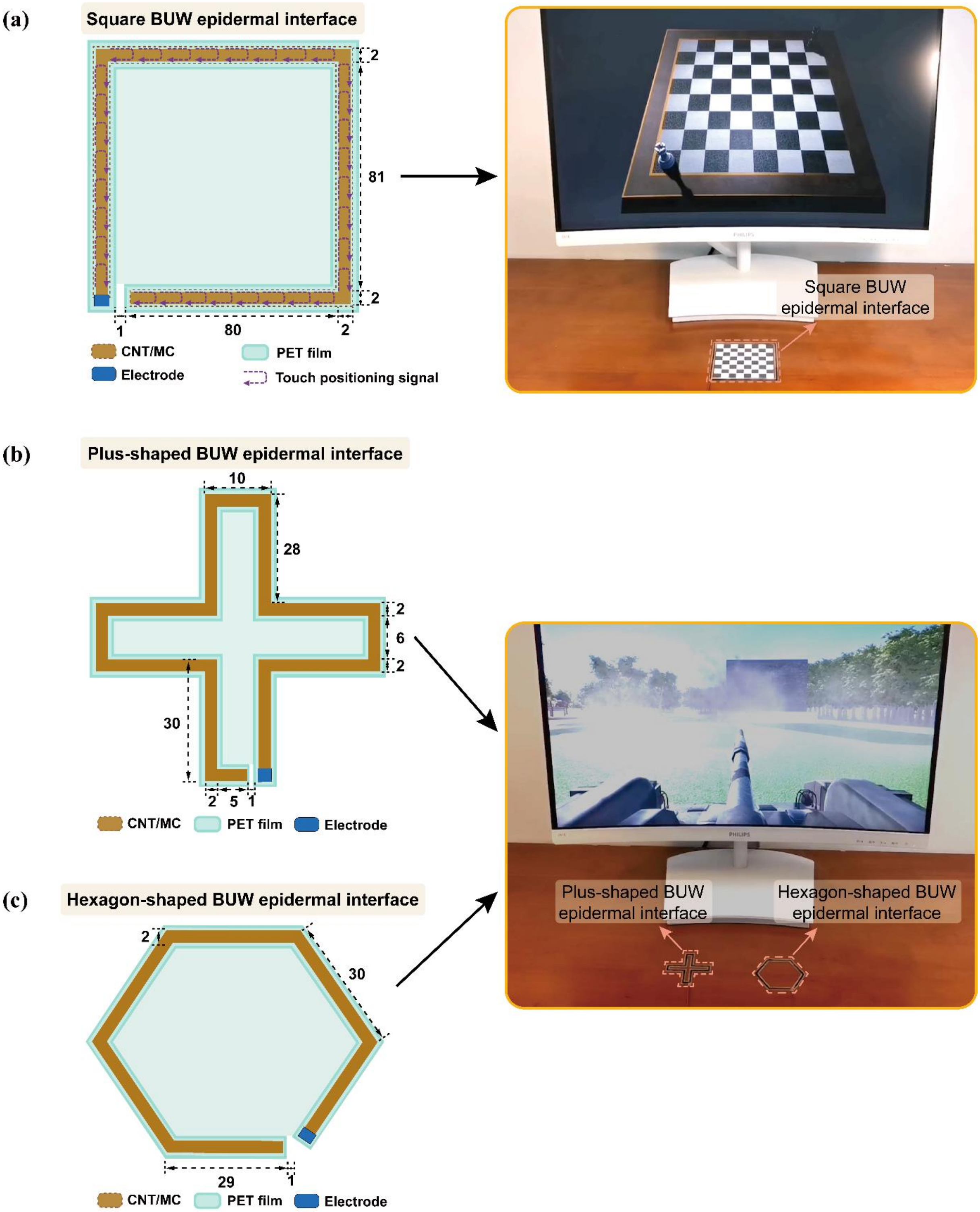

Fig. S16 Structural parameters of the **a** square, **b** plus-shaped, and **c** hexagon-shaped BUW epidermal interface. All the morphologies of the BUW epidermal interface were in top view. The unit in the figure was a millimeter

The square BUW epidermal interface was used for controlling the movement of the white and black chess. The plus-shaped and hexagon-shaped BUW epidermal interfaces were used for controlling virtual tank movement and the manipulation of the gun barrel.

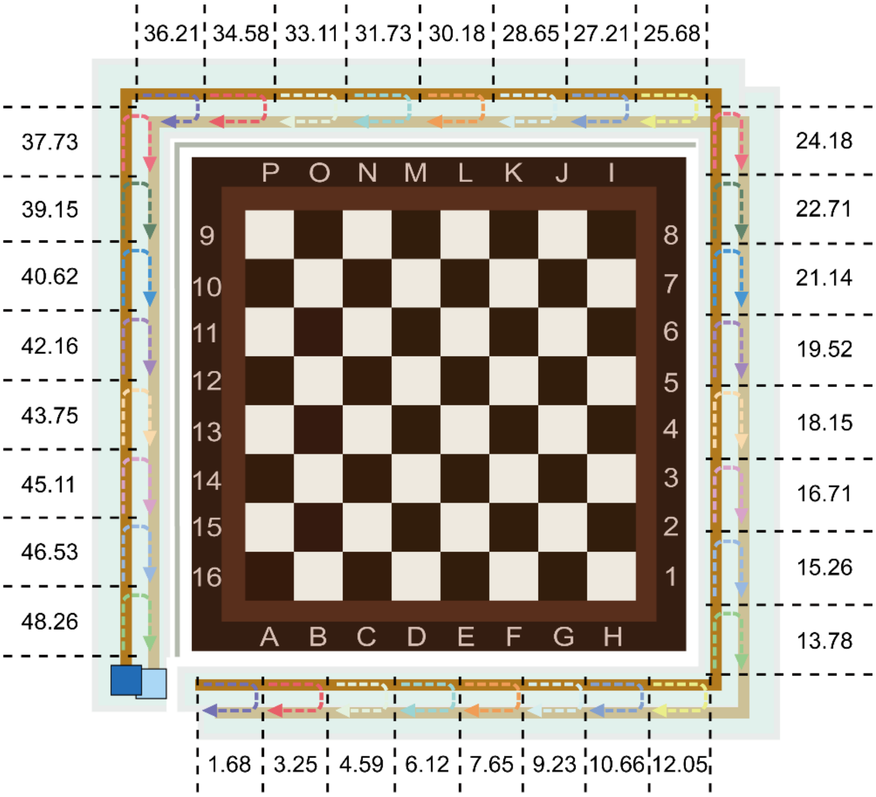

Fig. S17 Response resistance of each working segment of the BUW epidermal interface. The unit in the figure was kΩ
